# Cross-Validation Without Doing Cross-Validation in Genome-Enabled Prediction

**DOI:** 10.1534/g3.116.033381

**Published:** 2016-08-03

**Authors:** Daniel Gianola, Chris-Carolin Schön

**Affiliations:** * Department of Animal Sciences, University of Wisconsin-Madison, Wisconsin 53706; † Department of Dairy Science, University of Wisconsin-Madison, Wisconsin 53706; ‡ Department of Biostatistics and Medical Informatics, University of Wisconsin-Madison, Wisconsin 53706; § Department of Plant Sciences, Technical University of Munich School of Life Sciences, Technical University of Munich, Garching, Germany; ** Institute of Advanced Study, Technical University of Munich, Garching, Germany

**Keywords:** cross-validation, genomic selection, genomic prediction, genomic BLUP, reproducing kernels, GenPred, Shared Data Resources

## Abstract

Cross-validation of methods is an essential component of genome-enabled prediction of complex traits. We develop formulae for computing the predictions that would be obtained when one or several cases are removed in the training process, to become members of testing sets, but by running the model using all observations only once. Prediction methods to which the developments apply include least squares, best linear unbiased prediction (BLUP) of markers, or genomic BLUP, reproducing kernels Hilbert spaces regression with single or multiple kernel matrices, and any member of a suite of linear regression methods known as “Bayesian alphabet.” The approach used for Bayesian models is based on importance sampling of posterior draws. Proof of concept is provided by applying the formulae to a wheat data set representing 599 inbred lines genotyped for 1279 markers, and the target trait was grain yield. The data set was used to evaluate predictive mean-squared error, impact of alternative layouts on maximum likelihood estimates of regularization parameters, model complexity, and residual degrees of freedom stemming from various strengths of regularization, as well as two forms of importance sampling. Our results will facilitate carrying out extensive cross-validation without model retraining for most machines employed in genome-assisted prediction of quantitative traits.

Whole-genome-enabled prediction, introduced by Meuwissen *et al.* (2001), has received much attention in animal and plant breeding (*e.g.*, Van Raden 2008; Crossa *et al.* 2010; Lehermeier *et al.* 2013), primarily because it can deliver reasonably accurate predictions of the genetic worth of candidate animals or plants at an earlier time in the context of artificial selection. It has also been suggested for prediction of complex traits in human medicine (*e.g.*, de los Campos *et al.* 2011; Makowsky *et al.* 2011; Vázquez *et al.* 2012; López de Maturana *et al.* 2014; Spiliopoulou *et al.* 2015)

An important contribution of Utz *et al.* (2000) and of Meuwissen *et al.* (2001) was implanting cross-validation (CV) in plant and animal breeding as a mechanism for comparing prediction models, typically multiple linear regressions on molecular markers. In retrospect, it is perplexing that the progression of genetic prediction models, *e.g.*, from simple “sire” or “family” models in the late 1960s (Henderson *et al.* 1959) to complex multivariate and longitudinal specifications (Mrode 2014), proceeded without CV, as noted by Gianola and Rosa (2015). An explanation, at least in animal breeding, is the explosion of best linear unbiased prediction, BLUP (Henderson 1973, 1984). The power and flexibility of the linear mixed model led to the (incorrect) belief that a bigger model is necessarily better, simply because of extra explanatory power from an increasing degree of complexity. However, a growing focus on predictive inference and on CV has altered such perception. A simple prediction model may produce more stable, and even better, results than a complex hierarchical model (Takezawa 2006; Wimmer *et al.* 2013), and the choice can be made via CV. Today, CV is a *sine qua non* part of studies comparing genome-assisted prediction methods.

Most often, CV consists of dividing a data set with *n* cases (each including a phenotypic measurement and a vector of genomic covariables) into a number of folds (*K*) of approximately equal size. Data in *K* − 1 folds are used for model training, and to effect predictions of phenotypes in the testing fold, given the realized values of the genomic covariables. The prediction exercise is repeated for each fold, and the overall results are combined; this is known as a *K*-fold CV (Hastie *et al.* 2009). A loss function, such as mean-squared error (MSE) or predictive correlation is computed to gauge the various predictive machines compared. However, the process must be repeated a number of times, with folds reconstructed at random (whenever possible) to obtain measures of CV uncertainty (*e.g.*, Okut *et al.* 2011; Tusell *et al.* 2014). CV is computationally taxing, especially when Bayesian prediction models with a massive number of genomic covariates and implemented via Markov chain Monte Carlo (MCMC) are involved in the comparison.

Stylized formulae (*e.g.*, Daetwyler *et al.* 2008) suggest that the expected predictive correlation (“accuracy”) in genome-enabled prediction is proportional to training sample size (*n*). On intuitive grounds, more genetic variability ought to be spanned as a training sample grows, unless additional cases bring redundant information. With larger *n*, it is more likely that genomic patterns appearing in a testing set are encountered in model training. Although the formulae do not always fit real data well (Chesnais *et al.* 2016), it has been observed that a larger *n* tends to be associated with larger predictive correlations (Utz *et al.* 2000; Erbe *et al.* 2010).

Arguably, there is no better expectation than what is provided by a CV conducted under environmental circumstances similar to those under which the prediction machine is going to be applied. When *n* is small, the largest possible training set sample size is attained in a leave-one-out (LOO) CV, *e.g.*, Ober *et al.* (2015) with about 200 lines of *Drosophila melanogaster*. In LOO CV, *n* − 1 cases are used for model training, to then predict the single out-of-sample case. Model training involves *n* implementations, each consisting of a training sample of size *n* − 1 and a testing set of size 1.

It is not widely recognized that it is feasible to obtain CV results by running the model only once, which is well known for least-squares regression (*e.g.*, Seber and Lee 2003). Here, we show that this idea extends to other prediction machines, such as ridge regression (Hoerl and Kennard 1970), genome-based best linear unbiased prediction (GBLUP; Van Raden 2008), and reproducing kernel Hilbert spaces regression (RKHS; Gianola *et al.* 2006; Gianola and van Kaam 2008). It is also shown that the concept can be applied in a MCMC context to any Bayesian hierarchical model, *e.g.*, members of the “Bayesian alphabet” (Meuwissen *et al.* 2001; Gianola *et al.* 2009; Gianola 2013). This manuscript reviews available results for least-squares based CV, and shows how CV without actually doing CV can be conducted for ridge regression, BLUP of marker effects, GBLUP, and RKHS for any given kernel matrix. It is described how importance sampling can be used to produce Bayesian CV by running MCMC only once, which has great advantage in view of the intensiveness of MCMC computations. Illustrations are given by using a well characterized data set containing wheat grain yield as phenotype and 1279 binary markers as regressors, and the paper concludes with a *Discussion*. Most technical results are presented in a series of *Appendices*, to facilitate reading the main body of the manuscript.

## Cross-Validation with Ordinary Least-Squares

### Setting

A linear model used for regressing phenotypes on additive codes at biallelic marker loci (−1, 0, 1 for *aa*, *Aa* and *AA*, respectively), such as in a genome-wide association study, is

y=Xβ+e. (1)

Here, y is the n×1 vector of phenotypic measurements, $X=\left\{x_{ij}\right\}$ is the n×p marker matrix, and $x_{ij}$ is the number of copies of a reference allele at locus *j* observed in individual i; β is a p×1 vector of fixed regressions on marker codes, known as allelic substitution effects. Phenotypes and markers are often centered; if an intercept is fitted, the model is expanded by adding $\beta_{0}$ as an effect common to all phenotypes. The residual vector is assumed to follow the distribution $e∼N\left(0,I\sigma_{e}^{2}\right),$ where I is an n×n identity matrix, and $\sigma_{e}^{2}$ is a variance parameter common to all residuals.

The basic principles set here carry to the other prediction methods discussed in this paper. In this section, we assume rank(X)=p<n so that ordinary least-squares (OLS) or maximum likelihood under the normality assumption above can be used. The OLS estimator of β is $\hat{\beta }={\left(X\prime X\right)}^{-1}X\prime y$, and the fitted residual for datum *i* is $\hat{e_{i}}=y_{i}-x_{i}^{\prime }$ $\hat{\beta }$, where $x_{i}^{\prime }$ is the $i^{th}$ row of X. Assuming the model holds, $E\left(\hat{\beta }\right)=\beta$, so the estimator is unbiased. A review of the pertinent principles is given in *Appendix A*, from which we extract results.

It is shown in *Appendix A* that the uncertainty of a prediction, as measured by variance, increases with *p* (model complexity), and decreases with the size of the testing set, $n_{test}$. Two crucial matters in genome-enabled prediction must be underlined. First, if the model is unnecessarily complex, prediction accuracy (in the MSE sense) degrades unless the increase in variance is compensated by a reduction in prediction bias. Second, if the training set is made large at the expense of the size of the testing set, prediction mean squared error will be larger than otherwise. The formulae of Daetwyler *et al.* (2008) suggest that expected prediction accuracy, as measured by predictive correlation (not necessarily a good metric; González-Recio *et al.* 2014), increases with *n*. However, the variability of the predictions would increase, as found by Erbe *et al.* (2010) in an empirical study of Holstein progeny tests with alternative CV layouts. Should one aim at a higher expected predictive correlation or at a more stable set of predictions at the expense of the former? This question does not have a simple answer.

### Leave-one-out (LOO) cross-validation

LOO is often used when *n* is small and there is concern about the limited size of the training folds. Let $X_{\left[-i\right]}$ be X with its $i^{th}$ row $\left(x_{i}^{\prime }\right)$ removed, so that its order is (n−1)×p. Since

$$X^{\prime }X=\sum_{i=1}^{n}x_{i}{x^{\prime }}_{i},$$

$${X\prime }_{\left[-i\right]}X_{\left[-i\right]}=X\prime X-x_{i}{x^{\prime }}_{i}\text{;}\;\;i=1,2,\ldots ,\text{n},$$ (2)

are p×p matrices. Likewise, if $y_{\left[-i\right]}$ is y with its $i^{th}$ element removed, the OLS right-hand sides in LOO are

$${X\prime }_{\left[-i\right]}y_{\left[-i\right]}=\sum_{i=1}^{n}x_{i}y-x_{i}y_{i}=X^{\prime }y-x_{i}y_{i}$$ (3)

Making use of (81) in *Appendix B*, the least-squares estimator of β formed with the $i^{th}$ observation deleted from the model is expressible as

$$\begin{array}{c} {\hat{\beta }}_{\left[-i\right]}={\left({X\prime }_{\left[-i\right]}X_{\left[-i\right]}\right)}^{-1}{X\prime }_{\left[-i\right]}y_{\left[-i\right]}\\ =\left[{\left(X^{\prime }X\right)}^{-1}+\frac{{\left(X^{\prime }X\right)}^{-1}x_{i}x_{i}^{\prime }{\left(X^{\prime }X\right)}^{-1}}{1-x_{i}^{\prime }{\left(X^{\prime }X\right)}^{-1}x_{i}}\right]\left(X^{\prime }y-x_{i}y_{i}\right). \end{array}$$ (4)

Employing *Appendix C*, the estimator can be written in the form

$${\hat{\beta }}_{\left[-i\right]}=\hat{\beta }-\frac{{\left(X^{\prime }X\right)}^{-1}x_{i}\hat{e_{i}}}{1-h_{ii}},$$ (5)

where $h_{ii}=x_{i}^{\prime }{\left(X^{\prime }X\right)}^{-1}x_{i}$ and $\hat{e_{i}}=y_{i}-x_{i}^{\prime }\hat{\beta }$ is the fitted residual using all *n* observations in the analysis; the fitted LOO residual is

$$y_{i}-x_{i}^{\prime }{\hat{\beta }}_{\left[-i\right]}=\frac{y_{i}-x_{i}^{\prime }\hat{\beta }}{1-h_{ii}}.$$ (6)

Hence, the LOO estimator and prediction error can be computed directly from the analysis carried out with the entire data set: no need for *n* implementations.

Making use of (6), the realized LOO CV mean squared error of prediction is

$$\text{PMSE}\left(1\right)=\frac{1}{n}\sum_{i=1}^{n}{\left(\frac{y_{i}-x_{i}^{\prime }\hat{\beta }}{1-h_{ii}}\right)}^{2},$$ (7)

and the expected mean squared error of prediction is given by

$$\begin{array}{c} E_{y|X}\left[\text{PMSE}\left(1\right)\right]=\frac{1}{n}E\left[\sum_{i=1}^{n}{\left(\frac{y_{i}-x_{i}^{\prime }\hat{\beta }}{1-h_{ii}}\right)}^{2}\right]\\ =\frac{1}{n}\left\{\delta^{\prime }D\delta +tr\left[DVar\left(y-X\hat{\beta }\right)\right]\right\} \end{array}$$ (8)

where $D=\left\{{\left(1-h_{ii}\right)}^{-2}\right\}$ is an n×n diagonal matrix. As shown in *Appendix A* the LOO expected PMSE gives an upper bound for the expected squared error in least-squares based CV. The extent of overstatement of the error depends on the marker matrix X (via the $h^{\prime }s$) and on the prediction biases $\delta_{i}.$ Hence, LOO CV represents a conservative approach, with the larger variance of the prediction resulting from the smallest possible testing set size $\left(n_{\text{test}}=1\right)$ If the prediction is unbiased, the δ− terms vanish, and it is clear that observations with h− values closer to 1 contribute more to squared prediction error than those with smaller values, as the model is close to overfitting the former type of observations.

### Leave-d-out cross-validation

The preceding analysis suggests that reallocation of observations from training into testing sets is expected to reduce PMSE relative to the LOO scheme. Most prediction-oriented analyses use K− fold CV, where *K* is chosen arbitrarily (*e.g.*, K=2,5,10) as mentioned earlier; the decision of the number of folds is usually guided by the number of samples available. Here, we address this type of scheme generically by removing *d* out of the *n* observations available for training, and declaring the former as members of the testing set. Let $X_{\left[-d\right]}$ be X with *d* of its rows removed, and $y_{\left[-d\right]}$ be the data vector without the *d* corresponding data points. As shown in *Appendix D*,

$${\hat{\beta }}_{\left[-d\right]}=\hat{\beta }-{\left(X^{\prime }X\right)}^{-1}{X^{\prime }}_{\left[d\right]}{\left(I-H_{d}\right)}^{-1}{\hat{e}}_{\left[d\right]},$$ (9)

where $H_{d}=X_{\left[d\right]}{\left(X^{\prime }X\right)}^{-1}{X^{\prime }}_{\left[d\right]};$ note that ${\left(I-H_{d}\right)}^{-1}$ does not always exist but can be replaced by a generalized inverse, and ${\hat{\beta }}_{\left[-d\right]}$ will be invariant to the latter if p<n−d. Predictions are invariant with respect to the generalized inverse used. The similarity with (5) is clear: ${\left(I-H_{d}\right)}^{-1}$ appears in lieu of $1-h_{ii},$ ${X^{\prime }}_{\left[d\right]}{}_{}$ of order p×d contains (in columns) the rows of X being removed, and ${\hat{e}}_{\left[d\right]}=y_{\left[d\right]}-X_{\left[d\right]}\hat{\beta }$ are the residuals corresponding to the left out d−plet obtained when fitting the model to the entire data set.

The error of prediction of the *d* phenotypes entering into the testing set is

$$\begin{array}{c} y_{\left[d\right]}-X_{\left[d\right]}{\hat{\beta }}_{\left[-d\right]}=y_{\left[d\right]}-X_{\left[d\right]}\left\{\hat{\beta }-{\left(X^{\prime }X\right)}^{-1}{X^{\prime }}_{\left[d\right]}{\left(I-H_{d}\right)}^{-1}\left(y_{\left[d\right]}-X_{\left[d\right]}\hat{\beta }\right)\right\}\\ ={\left(I-H_{d}\right)}^{-1}{\hat{e}}_{\left[d\right]}. \end{array}$$ (10)

The mean-squared error of prediction of the *d* observations left out (testing set) becomes

$$\begin{array}{c} \text{PMSE}\left(d\right)=\frac{1}{d}{\left(y_{\left[d\right]}-X_{\left[d\right]}{\hat{\beta }}_{\left[-d\right]}\right)}^{\prime }\left(y_{\left[d\right]}-X_{\left[d\right]}{\hat{\beta }}_{\left[-d\right]}\right)\\ =\frac{1}{d}{{\hat{e}}^{\prime }}_{\left[d\right]}{\left(I-H_{d}\right)}^{-2}{\hat{e}}_{\left[d\right]}. \end{array}$$ (11)

The prediction bias obtained by averaging over all possible data sets is

$$E_{y_{\left[d\right]},y|,X,X_{\left[d\right]}}\left(y_{\left[d\right]}-X_{\left[d\right]}\hat{\beta }\right)=I\mu_{d}-X_{\left[d\right]}{\left(X^{\prime }X\right)}^{-1}X^{\prime }\mu =\delta_{d},$$ (12)

where $\mu_{d}$ is a d×1 vector of true means of the distribution of observations in the testing sets. After algebra,

$$Var_{y_{\left[d\right]},y|,X,X_{\left[d\right]}}\left(y_{\left[d\right]}-X_{\left[d\right]}\hat{\beta }\right)=\left(I-H_{d}\right)\sigma_{e}^{2}.$$ (13)

and

$$E_{y_{\left[d\right]},y|,X,X_{\left[d\right]}}\left[\text{PMSE}\left(d\right)\right]=\frac{1}{d}\left\{{\delta^{\prime }}_{d}{\left(I-H_{d}\right)}^{-2}\delta_{d}+tr\left[{\left(I-H_{d}\right)}^{-1}\right]\sigma_{e}^{2}\right\}.$$ (14)

Observe that the term in brackets is a matrix counterpart of (76) in *Appendix A*, with $H_{d}$ playing the role of $h_{ii}$ in the expression. The two terms in the equation above represent the contributions and bias and (co) variance to expected squared prediction error.

The next section illustrates how the preceding logic carries to regression models with shrinkage of estimated allelic substitution effects $\left(\hat{\beta }\right).$

## Cross-Validation with Shrinkage of Regression Coefficients

### BLUP of markers (ridge regression)

Assume again that phenotypes and markers are centered. Marker effects β are now treated as random variables and assigned the normal $N\left(0,I\sigma_{\beta }^{2}\right)$ distribution, where $\sigma_{\beta }^{2}$ is a variance component. The BLUP of β (Henderson 1975) is given by

$$\beta^{r}={\left(X^{\prime }X+I\lambda \right)}^{-1}X^{\prime }y,$$ (15)

where $\lambda =\frac{\sigma_{e}^{2}}{\sigma_{\beta }^{2}}$ is a shrinkage factor taken as known. BLUP has the same mathematical form as the ridge regression estimator (Hoerl and Kennard 1970), developed mainly for tempering problems caused by colinearity among columns of X in regression models where p<n, and with all regression coefficients likelihood-identified. The solution vector $\beta^{r}$ can also be assigned a Bayesian interpretation as a posterior expectation in a linear model with Gaussian residuals, and $N\left(0,I\sigma_{\beta }^{2}\right)$ used as prior distribution, with variance components known (Dempfle 1977; Gianola and Fernando 1986). A fourth view of $\beta^{r}$ is as a penalized maximum likelihood estimator under an $L_{2}$ penalty (Hastie *et al.* 2009). Irrespective of its interpretation, $\beta^{r}$ provides a “point statistic” of β for the n<p situation. In BLUP, or in Bayesian inference, it is not a requirement that the regression coefficients are likelihood identified. There is one formula with four interpretations (Robinson 1991).

Given a testing set with marker genotype matrix $X_{test},$ the point prediction of yet to be observed phenotypes is $X_{test}\beta^{r}.$ We consider LOO CV because subsequent developments assume that removal of a single case has a mild effect on $\sigma_{e}^{2}$ and $\sigma_{\beta }^{2}$. This assumption is reasonable for animal and plant breeding data sets where *n* is large, so removing a single observation should have a minor impact on, say, maximum likelihood estimates of variance components. If *λ* is kept constant, it is shown in *Appendix E* that

$$\beta_{\left[-i\right]}^{r}=\beta^{r}-\frac{C^{-1}x_{i}{\hat{e}}_{i}^{r}}{1-h_{ii}^{r}},$$ (16)

where $C=X^{\prime }X+I\lambda$, $h_{ii}^{r}={x^{\prime }}_{i}C^{-1}x_{i}$ and ${\hat{e}}_{i}^{r}=y_{i}-{x^{\prime }}_{i}\beta^{r}$ is the residual from ridge regression BLUP applied to the entire sample. A similar expression for leave-*d*-out cross-validation using the same set of variance components is also in *Appendix E*. If d/n is smaller and *n* is reasonably large, the error resulting from using variance components estimated from the entire data set should be small.

The error of predicting phenotype *i* is now $y_{i}-{x^{\prime }}_{i}\beta_{\left[-i\right]}^{r}$ and is expressible as

$$y_{i}-{x^{\prime }}_{i}\beta_{\left[-i\right]}^{r}=\frac{\left(y_{i}-{x^{\prime }}_{i}\beta^{r}\right)}{1-h_{ii}^{r}},$$ (17)

similar to that LOO OLS. Letting $\delta_{r}=\left\{E\left(y_{i}-{x^{\prime }}_{i}\beta^{r}\right)\right\}$ be a vector of prediction biases, $D_{r}=\left\{{\left(1-h_{ii}^{r}\right)}^{-2}\right\}$ and $M_{r}=I-XC^{-1}X^{\prime },$ the expected prediction MSE is

$$E_{y|X,\sigma_{e}^{2},\sigma_{\beta }^{2}}\left[{\text{PMSE}}_{r}\left(1\right)\right]=\frac{1}{n}{\delta^{\prime }}_{r}D_{r}\delta_{r}+\frac{\sigma_{e}^{2}}{n}tr\left[D_{r}M_{r}\left(XX^{\prime }\lambda^{-1}+I\right)M_{r}\right].$$ (18)

The first term is the average squared prediction bias, and the second is the prediction error variance. As $\sigma_{\beta }^{2}\to 0$, (18) tends to the least-squares PMSE(1).

### Genomic BLUP

Once marker effects are estimated as $\beta^{r}$, a representation of genomic BLUP (GBLUP) for *n* individuals is the n×1 vector $g=X\beta^{r}$ with its $i^{th}$ element being ${\hat{g}}_{i}={x^{\prime }}_{i}\beta^{r}$. In GBLUP, “genomic relationship matrices” are taken as proportional to $XX^{\prime }$ (where X often has centered columns); various genomic relationship matrices are in, *e.g.*, Van Raden (2008), Astle and Balding (2009) and Rincent *et al.* (2014). Using (16) LOO GBLUP (*i.e.*, excluding case *i* from the training sample) is

$${\hat{g}}_{\left[-i\right]}=X_{\left[-i\right]}\beta_{\left[-i\right]}^{r}=X_{\left[-i\right]}\beta^{r}-\frac{X_{\left[-i\right]}C^{-1}x_{i}\left(y_{i}-{x^{\prime }}_{i}\beta^{r}\right)}{1-h_{ii}}.$$ (19)

The formula above requires finding $\beta^{r},$ given λ; the procedure entails solving *p* equations on *p* unknowns and finding the inverse of C is impossible or extremely taxing when *p* is large. A simpler alternative based on the well-known equivalence between BLUP of marker effects and of additive genotypic value is used here.

If g=Xβ is a vector of marked additive genetic values and $\beta ∼N\left(0,I\sigma_{\beta }^{2}\right),$ then $g∼N\left(0,XX^{\prime }\sigma_{\beta }^{2}\right).$ Many genomic relationship matrices are expressible as $G=\frac{XX^{\prime }}{c}$ for some constant c, so that $g∼N\left(0,G\sigma_{g}^{2}\right)$ and $\sigma_{g}^{2}=c\sigma_{\beta }^{2}$ is called “genomic variance” or “marked additive genetic variance” if X encodes additive effects; clearly, there is no loss of generality if c=1 is used, thus preserving the *λ* employed for BLUP of marker effects. The model for the “signal” g becomes

y=Xβ+e=g+e. (20)

Letting $C=I+G^{-1}\lambda$, then $BLUP\left(g\right)=\hat{g}=\left\{{\hat{g}}_{i}\right\}=$ $C^{-1}y$ is GBLUP using all data points. *Appendix F* shows how LOO GBLUP and *d*-out GBLUP can be calculated indirectly from elements or blocks of C, and elements of y.

### RKHS regression

In RKHS regression (Gianola *et al.* 2006; Gianola and van Kaam 2008), input variables, *e.g.*, marker codes, can be transformed nonlinearly, potentially capturing both additive and nonadditive genetic effects (Gianola *et al.* 2014a, 2014b), as further expounded by Jiang and Reif (2015) and Martini *et al.* (2016). When a pedigree or a genomic relationship matrix is used as kernel, RKHS yields pedigree-BLUP and GBLUP, respectively, as special cases (Gianola and de los Campos 2008; de los Campos *et al.* 2009, 2010).

The standard RKHS model is

y=g+e=Kα+e, (21)

with g=Kα (and therefore $g_{i}=k_{i}^{\prime }\alpha$); K is an n×n positive (semi)definite symmetric matrix so that $K=K^{\prime };$ $\alpha =K^{-1}g$ when the inverse is unique, and $\alpha ∼N\left(0,K^{-1}\sigma_{\alpha }^{2}\right).$ BLUP(α) can be obtained by solving the system

$$\left(K^{2}+K\frac{\sigma_{e}^{2}}{\sigma_{\alpha }^{2}}\right)\hat{\alpha }=Ky,$$ (22)

with solution (since $K^{\prime }K=K^{2}$ and K is invertible)

$$\hat{\alpha }={\left(K+I\frac{\sigma_{e}^{2}}{\sigma_{\alpha }^{2}}\right)}^{-1}y.$$ (23)

The BLUP of g under a RKHS model is

$$\begin{array}{c} {\text{BLUP}}_{K}\left(g\right)={\text{BLUP}}_{K}\left(K\alpha \right)\\ =K{\left(K+I\frac{\sigma_{e}^{2}}{\sigma_{\alpha }^{2}}\right)}^{-1}y={\left(I+K^{-1}\lambda_{K}\right)}^{-1}y, \end{array}$$ (24)

where *K* stands for “kernel,” and $\lambda_{K}=\frac{\sigma_{e}^{2}}{\sigma_{\alpha }^{2}}$ . Putting $C_{K}^{-1}={\left(I+K^{-1}\lambda_{\text{RKHS}}\right)}^{-1},$ the RKHS solution ${\hat{g}}_{K}=C_{K}^{-1}y$ has the same form as $\text{BLUP}\left(g\right)=\hat{g}=$ $C^{-1}y,$ as given in the preceding section. Using *Appendix F*, it follows that

$${\tilde{g}}_{d,K}={\left(I-C_{K}^{dd}\right)}^{-1}\left({\hat{g}}_{d,K}-C_{K}^{dd}y_{d}\right),$$ (25)

$${\tilde{\epsilon }}_{d,K}=y_{d}-{\tilde{g}}_{d,K}={\left(I-C_{K}^{dd}\right)}^{-1}\left(y_{d}-{\hat{g}}_{d,K}\right),$$ (26)

and

$${\text{PMSE}}_{K}\left(d\right)=\frac{{\tilde{\epsilon }}^{\prime }{}_{d,K}{\tilde{\epsilon }}_{d,K}}{d}.$$ (27)

The previous expressions reduce to the LOO CV situation by setting d=1.

## Bayesian Cross-Validation

### Setting

Many Bayesian linear regression on markers models have been proposed for genome-assisted prediction of quantitative traits (*e.g.*, Meuwissen *et al.* 2001; Heslot *et al.* 2012; de los Campos *et al.* 2013; Gianola 2013). All such models pose the same specification for the Bayesian sampling model (a linear regression), but differ in the prior distribution assigned to allelic substitution effects. Implementation is often via MCMC, where computations are intensive even in the absence of CV; shortcuts and approximations are not without pitfalls. Is it possible to do CV by running an MCMC implementation only once? What follows applies both to LOO and d− out CV situations as well as to any member of the Bayesian alphabet (Gianola *et al.* 2009; Gianola 2013)

Suppose some Bayesian model has been run with MCMC, leading to *S* samples collected from a distribution with posterior density p(θ|y,H); here, θ are all unknowns to be inferred and *H* denotes hyper-parameters arrived at in a typically subjective manner, *e.g.*, arbitrary variances in a four-component mixture distribution assigned to substitution effects (MacLeod *et al.* 2014). In CV, the data set is partitioned into $y=\left(y_{test},y_{train}\right)$, training and testing sets are chosen according to the problem in question, and Bayesian learning is based on the posterior distribution $\left[\theta |y_{train},H\right]$. Predictions are derived from the predictive distribution of the testing set data

$$p\left(y_{test}|y_{train},H\right)=\int p\left(y_{test}|\theta \right)p\left(\theta |y_{train},H\right)d\theta ;$$ (28)

the preceding assumes that $y_{test}$ is independent of $y_{train}$ given θ_,_ a standard assumption in genome-enabled prediction. The point predictor chosen most often is the expected value of the predictive distribution

$$\begin{array}{c} E\left(y_{test}|y_{train},H\right)=\int \int y_{test}p\left(y_{test}|\theta \right)p\left(\theta |y_{train},H\right)d\theta dy_{test}\\ =\int \int y_{test}p\left(y_{test},\theta |y_{train},H\right)d\theta dy_{test} \end{array}$$ (29)

$$=\int E\left(y_{test}|\theta \right)p\left(\theta |y_{train},H\right)d\theta$$ (30)

In the context of sampling, representation (29) implies that one can explore the “augmented” distribution $\left[y_{test},\theta |y_{train},H\right]$, and estimate $E\left(y_{test}|y_{train},H\right)$ by ergodic averaging of $y_{test}$ samples. Representation (30) uses Rao-Blackwellization: if $E\left(y_{test}|\theta \right)$ can be written in closed form, as is the case for regression models $\left(X_{test}\beta \right)$, the Monte Carlo variance of an estimate of $E\left(y_{test}|y_{train},H\right)$ based on (30) is less than, or equal to, that of an estimate obtained with (29).

We describe Bayesian LOO CV, but extension to a testing set of size *d* is straightforward. In LOO, the data set is partitioned as $y=\left(y_{i},y_{-i}\right),$ i=1,2,…,n, where $y_{i}$ is the predictand and $y_{-i}$ is the vector containing all other phenotypes in the data set. A brute force process involves running the Bayesian model *n* times, producing the posterior distributions $\left[\theta |y_{-i},H\right];$ i=1,2,…,n. Since LOO CV is computationally formidable in an MCMC context, procedures based on drawing samples from [θ|y,H] and converting these into realizations from $\left[\theta |y_{-i},H\right]$ can be useful (*e.g.*, Gelfand *et al.* 1992; Gelfand 1996; Vehtari and Lampinen 2002). Use of importance sampling, and of sampling importance resampling (SIR), algorithms for this purpose is discussed next. Cantet *et al.* (1992) and Matos *et al.* (1993) present early applications of importance sampling to animal breeding.

### Importance sampling

We seek to estimate the mean of the predictive distribution of the left-out data point $E\left(y_{i}|y_{-i},H\right).$ Since $e_{i}∼N\left(0,\sigma_{e}^{2}\right)$ is independent of $y_{-i}$, one has

$$E\left(y_{i}|y_{-i},H\right)=x_{i}^{\prime }E\left(\beta |y_{-i},H\right).$$ (31)

As shown in *Appendix G*

$$E\left(\beta |y_{-i},H\right)=\frac{E_{\beta |y,H}\left[w_{i}\left(\beta \right)\beta \right]}{E_{\beta |y,H}\left[w_{i}\left(\beta \right)\right]},i=1,2,\ldots ,n.$$ (32)

Here, $w_{i}\left(\beta \right)=\frac{p\left(\beta |y_{-i},H\right)}{p\left(\beta |y,H\right)}$ is called an “importance sampling” weight (Gelfand *et al.* 1992; Albert 2009). Expression (32) implies that the posterior mean of β in a training sample can be expressed as the ratio of the posterior means of w(β)β, and of w(β) taken under a Bayesian run using the entire data set. It is shown in *Appendix F* that, given draws $\beta^{\left(s\right)},$ $\sigma_{e}^{2\left(s\right)}$ (s=1,2,…,S) from the full-posterior distribution, the posterior expectation can in equation (32) be estimated as

$$\hat{E}\left(\beta |y_{-i},H\right)=\sum_{s=1}^{S}w_{i,s}\beta^{\left(s\right)};\;i=1,2,\ldots ,n,$$ (33)

where

$$w_{i,s}=\frac{p^{-1}\left(y_{i}|\beta^{\left(s\right)},\sigma_{e}^{2\left(s\right)}\right)}{\sum_{s=1}^{S}p^{-1}\left(y_{i}|\beta^{\left(s\right)},\sigma_{e}^{2\left(s\right)}\right)};\;i=1,2,\ldots ,n;\;\;s=1,2,\ldots ,S.$$ (34)

By making reference to (31), it turns out that a Monte Carlo estimate of the mean of the predictive distribution of datum *i* in the Bayesian LOO CV is given by

$$\hat{E}\left(y_{i}|y_{-i},H\right)=x_{i}^{\prime }\sum_{s=1}^{S}w_{i,s}\beta^{\left(s\right)};\;i=1,2,\ldots ,n.$$ (35)

This type of estimator holds for any Bayesian linear regression model irrespective of the prior adopted, *i.e.*, it is valid for any member of the “Bayesian alphabet” (Gianola *et al.* 2009; Gianola 2013). In d−out CV, the prediction is

$$\hat{E}\left(y_{d}|y_{\left[-d\right]},H\right)=X_{d}\sum_{s=1}^{S}w_{d}\beta^{\left(s\right)},$$ (36)

where

$$w_{d}=\frac{p^{-1}\left(y_{d}|\beta^{\left(s\right)},\sigma_{e}^{2\left(s\right)}\right)}{\sum_{s=1}^{S}p^{-1}\left(y_{d}|\beta^{\left(s\right)},\sigma_{e}^{2\left(s\right)}\right)},$$ (37)

where $p\left(y_{d}|\beta^{\left(s\right)},\sigma_{e}^{2\left(s\right)}\right)=\prod_{j=1}^{d}p^{-1}\left(y_{j}|\beta^{\left(s\right)},\sigma_{e}^{2\left(s\right)}\right)$ for *j* being a member of the d−plet of observations forming the testing set.

The importance sampling weights are the reciprocal of conditional likelihoods; this specific mathematical representation can produce imprecise estimates of posterior expectations, especially if the posterior distribution with all data has much thinner tails than the posterior based on the training set. Vehtari and Lampinen (2002) calculate the “effective sample size” for a LOO CV as

$$S_{eff,i}=\frac{1}{\sum_{s=1}^{S}w_{i,s}^{2}}=\frac{1}{S\left[Var\left(w_{i}\right)+{\bar{w_{i}}}^{2}\right]}.$$ (38)

If all weights are equal over samples, the weight assigned to any draw is $S^{-1},$ and the variance of the weights is 0, yielding $S_{eff,i}=S;$ on the other hand, $S_{eff,i}$ can be much smaller than *S* if the variance among weights is large, *e.g.*, when some weights are much larger than others.

The SIR algorithm described by Rubin (1988), Smith and Gelfand (1992) and Albert (2009) can be used to supplement importance sampling; SIR can be viewed as a weighted bootstrap. Let the sampled values and the (normalized) importance sampling weights be $\beta^{\left(s\right)}$ and $w_{i,s},$ respectively, for i=1,2,…,n and s=1,2,…,S. Then, obtain a resample of size *S* by sampling with replacement over $\beta^{\left(1\right)},\beta^{\left(2\right)},\ldots ,\beta^{\left(S\right)}$ with unequal probabilities proportional to $w_{i,1},w_{i,2},\ldots ,w_{i,S},$ respectively, obtaining realizations $\beta_{rep}^{\left(1\right)},\beta_{rep}^{\left(2\right)},\ldots ,\beta_{rep}^{\left(S\right)}.$ Finally, average realizations for estimating $E\left(\beta |y_{-i},H\right)$ in (31).

### The special case of “Bayesian GBLUP”

The term “Bayesian GBLUP” is unfortunate but has become entrenched in animal and plant breeding. It refers to a linear model that exploits genetic or genomic similarity matrix among individuals (as in GBLUP), but where the two variance components are unknown and learned in a Bayesian manner. Prior distributions typically assigned to variances are scale inverted chi-square processes with known scale and degrees of freedom parameters (*e.g.*, Pérez and de los Campos 2014). The model is y=g+e, with $e∼N\left(0,I\sigma_{e}^{2}\right)$; the hierarchical prior is $g|\sigma_{g}^{2}∼N\left(0,G\sigma_{g}^{2}\right);$ $\sigma_{g}^{2}|S_{g}^{2},\nu_{g}$ and $\sigma_{e}^{2}|S_{e}^{2},\nu_{e}$, where the hyper-parameters are $H=\left(S_{g}^{2},\nu_{g},S_{e}^{2},\nu_{e}\right)$. A Gibbs sampler iteratively loops over the conditional distributions

$$\begin{array}{c} g|ELSE∼N\left(\hat{g},V_{g|ELSE}\right),\\ \sigma_{g}^{2}|ELSE∼\left(g^{\prime }G^{-1}g+S_{g}^{2}\nu_{g}\right)\chi_{n+\nu_{g}}^{-2},\\ \sigma_{e}^{2}|ELSE∼\left[{\left(y-g\right)}^{\prime }\left(y-g\right)+S_{e}^{2}\nu_{e}\right]\chi_{n+\nu_{e}}^{-2}. \end{array}$$ (39)

Above, ELSE denotes the data plus *H* and all other parameters other than the ones being sampled in a specific conditional posterior distribution; $\chi_{df}^{-2}$ indicates the reciprocal of a draw from a central chi-square distribution on df degrees of freedom. The samples of g are drawn from a multivariate normal distribution of order n. Its mean, $\hat{g},$ is GBLUP computed at the current state of the variance ratio, which varies at random from iteration to iteration; the covariance matrix is $V_{g|ELSE}=C^{-1}\sigma_{e}^{2}.$ The current GBLUP is calculated as $\hat{g}=C^{-1}y=$ ${\left(I+G^{-1}\lambda \right)}^{-1}y;$ in this representation, $G^{-1}$ must exist. If it does not, a representation of GBLUP that holds is

$$\hat{g}=G{\left(G+I\lambda \right)}^{-1}y=By,$$ (40)

where B is an n×n matrix of regression coefficients of g on y. Likewise

$$V_{g|ELSE}=G{\left(G+I\lambda \right)}^{-1}\sigma_{e}^{2}=B\sigma_{e}^{2}.$$ (41)

Once the Gibbs sampler has been run and burn-in iterations discarded, *S* samples become available for posterior processing, with sample *s* consisting of $\left\{g_{1}^{\left(s\right)},g_{2}^{\left(s\right)},\ldots ,g_{n}^{\left(s\right)},\sigma_{g}^{2\left(s\right)},\sigma_{e}^{2\left(s\right)}\right\}.$ In a leave-*d*-out CV, the posterior expectation of $g_{j}$ (the point predictor of the future phenotype of individual *j*) is estimated as

$$\hat{E}\left(g_{j}|y_{\left[-d\right]},H\right)=\sum_{s=1}^{S}w_{j,s}g_{2}^{\left(s\right)};j=1,2,\ldots ,n,$$ (42)

where

$$w_{j,s}=\frac{p^{-1}\left(y_{d}|g_{d}^{\left(s\right)},\sigma_{e}^{2\left(s\right)}\right)}{\sum_{s=1}^{S}p^{-1}\left(y_{d}|g_{d}^{\left(s\right)},\sigma_{e}^{2\left(s\right)}\right)};\;\;\;i=1,2,\ldots ,n;\;\;\;s=1,2,\ldots ,S,$$ (43)

and

$$p\left(y_{d}|g_{d}^{\left(s\right)},\sigma_{e}^{2\left(s\right)}\right)=\prod_{d\in Test}\frac{1}{\sqrt{2\pi \sigma_{e}^{2\left(s\right)}}}\text{exp}\left[-\frac{{\left(y_{d}-g_{d}^{\left(s\right)}\right)}^{2}}{2\sigma_{e}^{2\left(s\right)}}\right],$$ (44)

with the product of the normal densities taken over members of the testing set. Sampling weights may be very unstable if *d* is large.

### Data availability

The authors state that all data necessary for confirming the conclusions presented in the article are represented fully within the article.

## Evaluation of Methodology

The methods described here were evaluated using the wheat data available in package BGLR (Pérez and de los Campos 2014). This data set is well curated and has also been used by, *e.g.*, Crossa *et al.* (2010), Gianola *et al.* (2011) and Long *et al.* (2011). The data originated in trials conducted by the International Maize and Wheat Improvement Center (CIMMYT), Mexico. There are 599 wheat inbred lines, each genotyped with 1279 DArT (Diversity Array Technology) markers and planted in four environments; the target trait was grain yield in environment 1. Sample size was then n=599 with p=1279 being the number of markers. These DArT markers are binary (0,1) and denote presence or absence of an allele at a marker locus in a given line. There is no information on chromosomal location of markers. The objective of the analysis was to illustrate concepts, as opposed to investigate a specific genetic hypothesis. The data set of moderate size allowed extensive replication and reanalysis under various settings.

### LOO *vs.* leave-d-out CV: ordinary least-squares

The linear model had an intercept and regressions on markers 301 through 500 in the data file; markers were chosen arbitrarily. Here, p=201 and n=599, ensuring unique OLS estimates of substitution effects, *i.e.*, there was no rank deficiency in X.

Seven CV layouts were constructed in which testing sets of sizes 2, 3,…, 7, or 8 lines were randomly drawn (without replacement) from the 599 inbred lines. Training set sizes decreased accordingly, *e.g.*, for d=7, training sample size was 599 – 7= 592. Larger sizes of testing sets were not considered because $\left(I-H_{d}\right)$ in (9) became singular as *d* increased beyond that point. The training-testing process was repeated 300 times at random, to obtain an empirical distribution of prediction mean squared errors.

**Figure 1:**
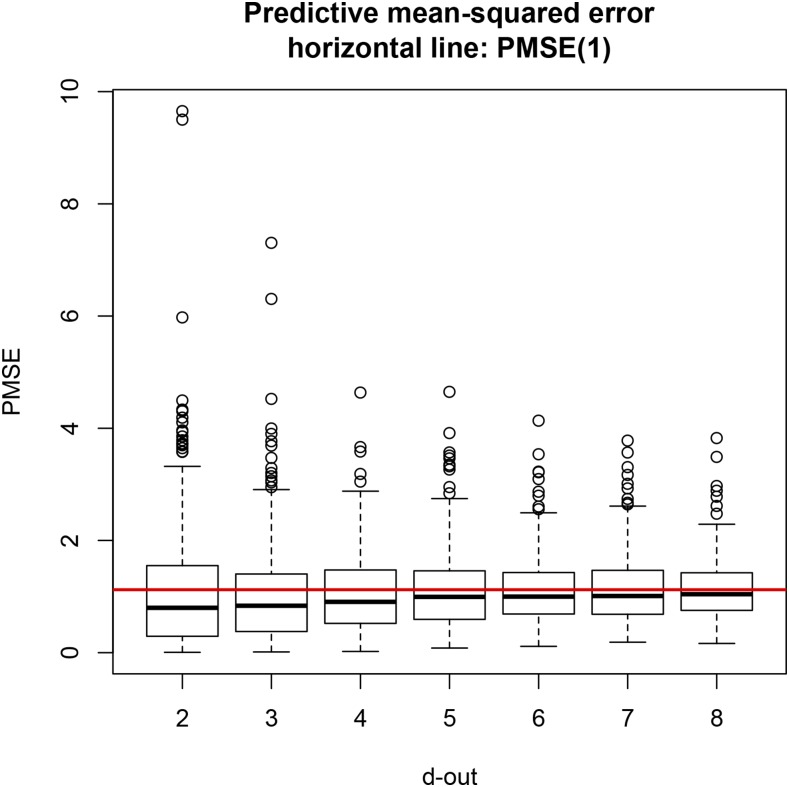
Predictive mean squared error (PMSE) of ordinary least-squares for seven cross-validation (CV) layouts, each replicated 300 times at random. Training sets had size 599 – *d* (*d* = 2,3,…,7, and 8). Horizontal line is PMSE for leave-one-out CV.

For LOO CV, regression coefficients were calculated using (5), and the predictive mean squared error was computed as in (7). For the leave-*d*-out CV, regressions and PMSE were computed with (9) and (11), respectively. Figure 1 shows that the median PMSE for leave-*d*-out CV was always smaller than the LOO PMSE (horizontal line), although it tended toward the latter as *d* increased, possibly due to the increasingly smaller training sample size. PMSE in LOO was 1.12, while it ranged from 0.80 to 1.04 for testing sets containing two or more lines. An increase in testing set size at the expense of some decrease in training sample size produced slightly more accurate but less variable predictions (less spread in the distribution of PMSE); this trend can be seen in the box plots depicted in Figure 1. Differences were small but LOO was always less accurate.

### BLUP of marker effects

The developments for ridge regression or BLUP of marker effects depend on assuming that allocation of observations into testing sets, with a concomitant decrease in training set size, does not affect the ratio of variance components appreciably.

First, we examined consequences of removing each of the 599 lines at a time on maximum likelihood estimates (MLE) of marker $\left(\sigma_{\beta }^{2}\right)$ and residual $\left(\sigma_{e}^{2}\right)$ variances. The model was as in (1), without an intercept (phenotypes were centered), assuming $\beta ∼N\left(0,I\sigma_{\beta }^{2}\right)$ and $e∼N\left(0,I\sigma_{e}^{2}\right)$ were independently distributed, and with all 1279 markers used when forming $XX^{\prime }.$ An eigen-decomposition of $XX^{\prime }$ coupled with the *R* function optim (G. de los Campos, personal communication) was used for computing MLE. It was assumed that convergence was always to a global maximum, as it was not practical to monitor the 599 implementations for convergence in each case. MLE of $\lambda =\frac{\sigma_{e}^{2}}{\sigma_{\beta }^{2}}$ were found by replacing the unknown variances by their corresponding estimates.

**Figure 2:**
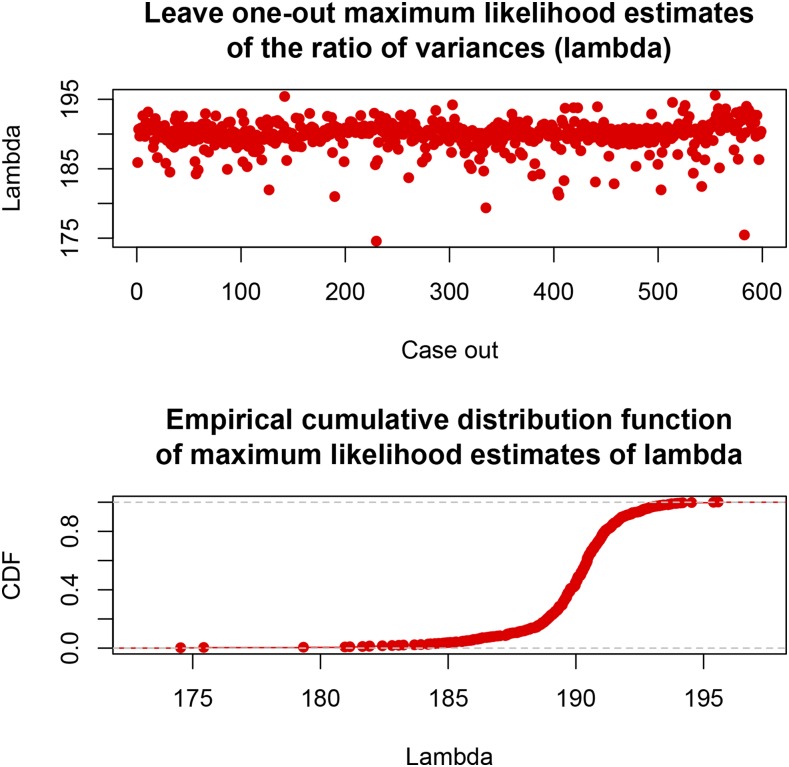
Maximum likelihood estimates of $\lambda =\frac{\sigma_{e}^{2}}{\sigma_{\beta }^{2}}$ for each of 599 leave-one-out settings; $\sigma_{e}^{2}=$ residual variance, $\sigma_{\beta }^{2}=$ variance of marker effects. The bottom panel gives the empirical distribution function of the estimates.

With all lines used in the analysis, MLE(λ)=189.9. Figure 2 displays the 599 estimates of *λ*, and the resulting empirical cumulative distribution function when LOO was used. Removal of a single line produced MLE of *λ* ranging from 174.5 to 195.6 (corresponding to estimates of $\sigma_{\beta }^{2}$ spanning the range $5.1-5.7\times 10^{-3}$); the 5 and 95 percentiles of the distribution of the LOO estimates of *λ* were 185.9 and 192.7, respectively. Model complexity (Ruppert *et al.* 2003; Gianola 2013) was gauged by evaluating the “effective number of parameters” as $p_{\text{eff}}=tr\left[X{\left(X^{\prime }X+I\lambda \right)}^{-1}X^{\prime }\right]$; the “effective degrees of freedom” are $\nu_{e}=n-p_{\text{eff}}.$ For the entire data set with n=599 and p=1279, variation of *λ* from 174.5 to 195.6 was equivalent to reducing $p_{\text{eff}}$ from 164.2 to 155.3, with $\nu_{e}$ ranging from 435.8 to 443.7. These metrics confirm that the impact of removing a single line from the training process was fairly homogeneous across lines.

**Figure 3:**
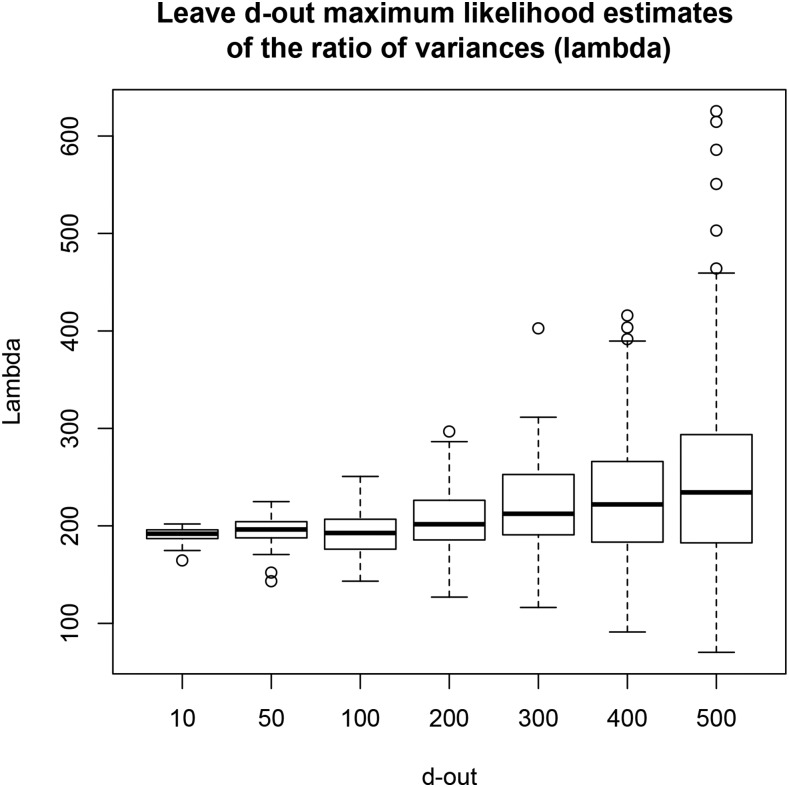
Maximum likelihood estimates of $\lambda =\frac{\sigma_{e}^{2}}{\sigma_{\beta }^{2}}$ for each of seven CV settings, each replicated 100 times at random. Training sets had size 599 – *d*; *d* = 10, 50, 100, 200, 300, 400, and 500.

**Figure 4:**
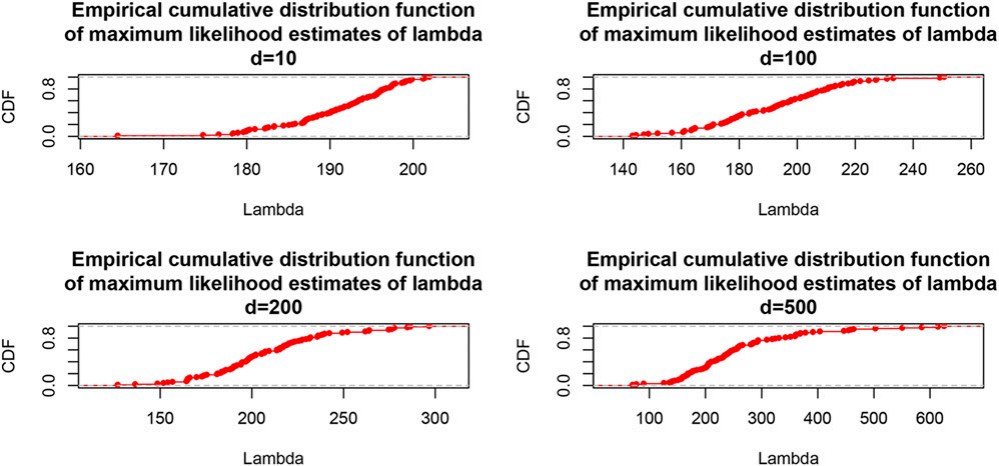
Empirical distribution function of maximum likelihood estimates of $\lambda =\frac{\sigma_{e}^{2}}{\sigma_{\beta }^{2}}$ for each of four CV settings each replicated 100 times at random $;\;\sigma_{e}^{2}=$ residual variance, $\sigma_{\beta }^{2}=$ variance of marker effects. Training set sizes were 599 – *d*; *d* = 10, 100, 200, and 500.

Next, we excluded d= 10, 50, 100, 200, 300, 400, and 500 lines from the analysis, while keeping p=1279 constant. The preceding was done by sampling with replacement the appropriate number of rows from the entire data matrix (y,X), and removing these rows from the analysis; the procedure was repeated 100 times at random for each value of d, to obtain an empirical distribution of the MLE. Figure 3 and Figure 4 depict the distributions of estimates. As *d* increased (training sample size decreased) the median of the estimates and their dispersion increased. Medians were 192.0 (d=10), 196.3 (d=50), 192.7 (d=100), 201.7 (d=200), 212.5 (d=300), 222.0 (d=400), and 234.3 (d=500). The increase of medians as training sample size decreased can be explained as follows: (a) stronger shrinkage (larger λ) must be exerted on marker effects to learn signal from 1279 markers as sample size decreases. (b) MLE of variance components have a finite sample size bias, which might be upwards for *λ* here; bias cannot be measured, so the preceding is conjectural.

In short, it appears that keeping *λ* constant in a LOO setting is reasonable; however, the estimated variance ratio was sensitive with respect to variation in training set size when 100 or more lines, *i.e.*, 15% or more of the total number of cases, were removed for model training.

**Figure 5:**
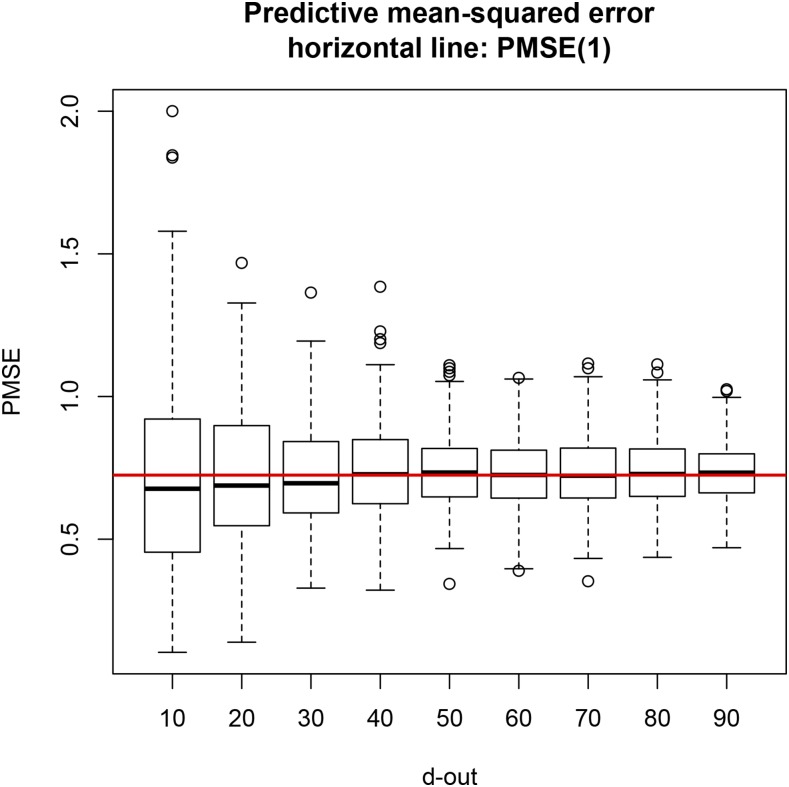
PMSE of BLUP of markers (ridge regression) for 300 testing sets in each of nine CV settings of sizes *d* = 10, 20, 30, 40, 50, 60, 70, 80, and 90; training set size was 599 – *d*.

To assess the impact on PMSE of number of lines removed from training and allocated to testing, 300 testing sets for each of d=10,20,…,80,90 lines were formed by randomly sampling (with replacement) from the 599 lines. The regression model was trained on the remaining lines using the entire battery of 1279 markers with λ=190. Figure 5 shows the distribution of PMSE for each of the layouts. A comparison with Figure 1 shows that the PMSEs for BLUP were smaller than for OLS; this was expected because, even though training set sizes were smaller than those used for OLS, BLUP predictions with λ=190 are more stable and the model was more complex, since 1279 markers were fitted jointly. As testing set size increased, median PMSE was 0.68 (d=10) 0.70 (d=20) and 0.72–0.73 for the other testing set sizes. For LOO, PMSE was 0.72. As in the case of OLS, the distribution of PMSE over replicates became narrower as *d* grew. As anticipated, decreases in training set size produced a mild deterioration in accuracy of prediction (in an MSE sense) but generated a markedly less variable CV distribution. Testing sets of about 10% of all lines produced a distribution of PMSE with a similar spread to what was obtained with larger testing sets without sacrificing much in mean accuracy. We corroborated that attaining the largest possible training sample is not optimal if done at the expense of testing set size, because predictions are more variable.

**Figure 6:**
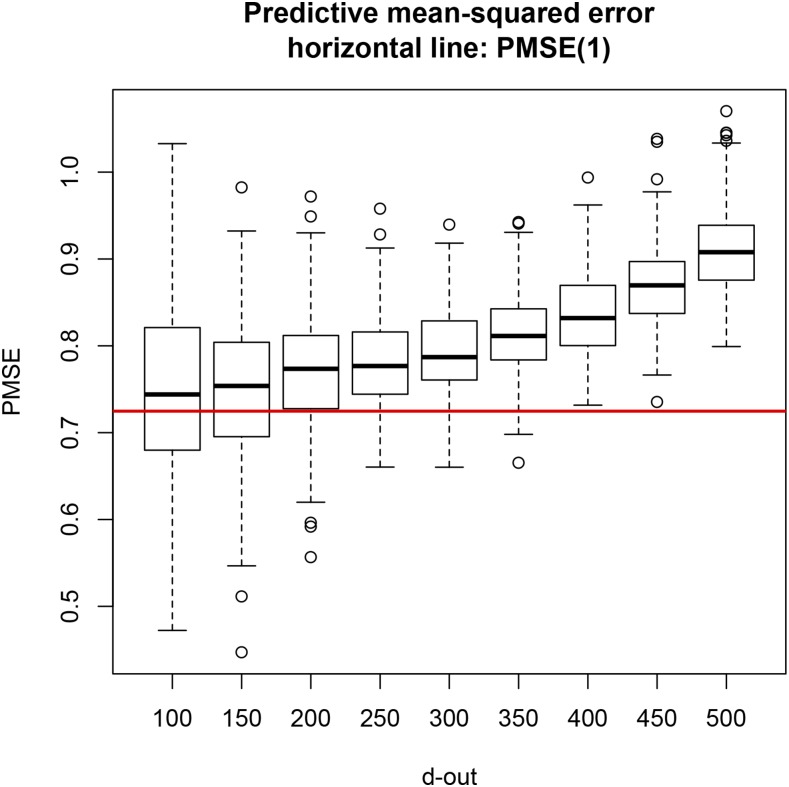
PMSE of BLUP of markers (ridge regression) for 300 testing sets in each of nine CV settings of sizes *d* = 100, 150, 200, 250, 300, 350, 400, 450, and 500; training set size was 599 – *d*.

**Figure 7:**
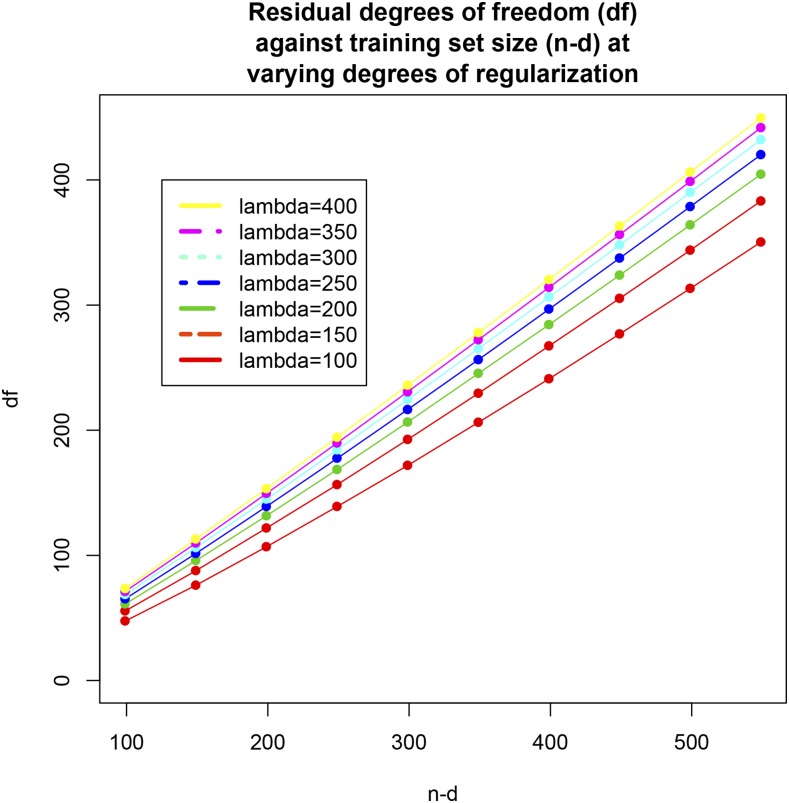
Effective residual degrees of freedom against training sets sizes (*n* – *d* = 99, 149, 199, 249, 299, 349, 399, 449, 499, and 549) at selected values of the regularization parameter (*λ* = 100,150, 200, 250, 300, 350, and 400). Values are averages of 50 random replications.

**Figure 8:**
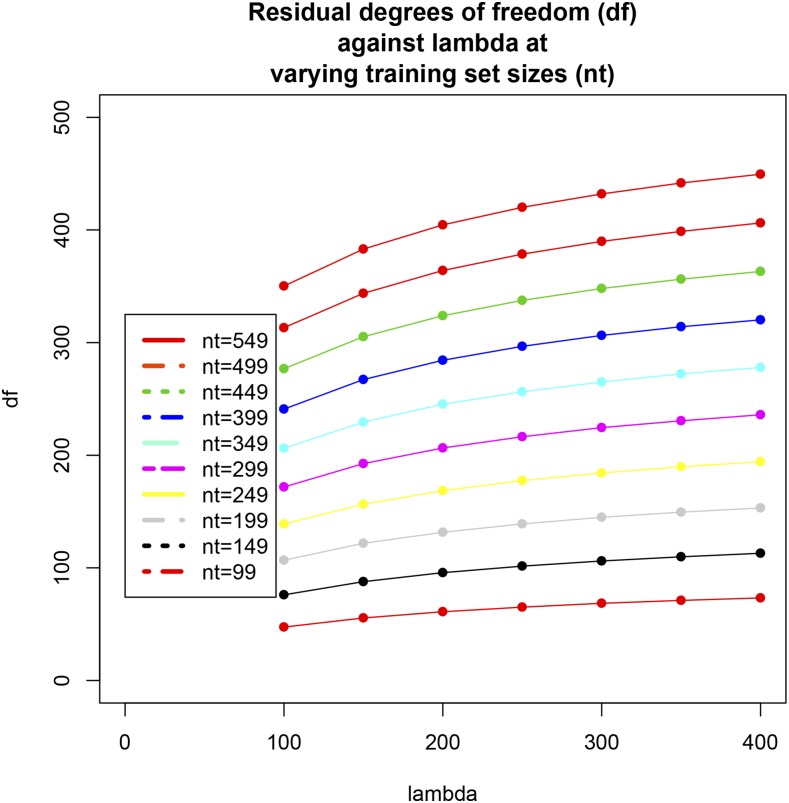
Effective residual degrees of freedom against the regularization parameter (*λ* = 100, 150, 200, 250, 300, 350, and 400) at various training sets sizes (*n* – *d* = 99, 149, 199, 249, 299, 349, 399, 449, 499, 549). Values are averages of 50 random replications.

Testing sets of size d=100 to d=500 (in increments of 50 lines) were evaluated as in the preceding case, again using 300 replicates for each setting and with λ=190. Comparison of Figure 6 with Figure 5 indicates that a marked deterioration in PMSE ensued, which may be due to insufficient regularization or overfitting from the decrease in training set size. For example, a testing set of size 500 implies that the model with 1279 markers was trained using only 99 inbred lines. In this case, stronger regularization (shrinkage of regression coefficients toward 0) may be needed than what is effected by λ=190. To examine whether overfitting or insufficient regularization was the source of degradation in PMSE, the effective residual degrees of freedom

$$\nu_{e}=n-d-tr\left[X_{\left[-d\right]}{\left({X\prime }_{\left[-d\right]}X_{\left[-d\right]}+I\lambda \right)}^{-1}{X\prime }_{\left[-d\right]}\right]$$ (45)

were calculated for each of 70 combinations of d=(50,100,…,450,500) and λ=(100,150,…,350,400); each combination was replicated 50 times by sampling with replacement from (y,X) and extracting the appropriate number of rows . The $\nu_{e}$ values were averaged over the 50 replications. Figure 7 displays $\nu_{e}$ plotted against training set size (n−d= 99, 149,…,499, 549): the impact of variations in *λ* on $\nu_{e}$ was amplified in absolute and relative terms as training set size increased. For instance, for n−d=99, each observation in the training set contributed 0.48 and 0.74 residual degrees of freedom when *λ* varied from 100 to 400; when n−d=549, the corresponding contributions were 0.64 and 0.82. Figure 8 shows how $\nu_{e}$ varied with *λ* for each of the training set sizes. Overfitting did not seem to be the cause of degradation in PMSE because the models “preserved” a reasonable number of degrees of freedom in each case considered.

These results reinforce the point that, in shrinkage-based methods, such as GBLUP or any member of the “Bayesian alphabet” (Gianola *et al.* 2009; Gianola 2013), there is an interplay between sample size, model complexity, and strength of regularization. The effective number of parameters in the training process is given by $n-d-\nu_{e},$ and here it varied from 25.64 (n−d=99,λ=400) to 198.73 (n−d=549,λ=100). Even though p=1279 markers were fitted, the model was not able to estimate beyond about 200 linear combinations of marker effects. This illustrates that the “prior” (*i.e.*, the distribution assigned to marker effects) matters greatly when n≪p . In other words, there were ∼ 1079 estimates of marker effects that are statistical artifacts from regularization, and which should not be construed as sensible estimates of marker locus effects, as pointed out by Gianola (2013). Bayesian learning would gradually improve over time if *n* would grow faster than p, which seems unlikely given a tendency toward overmodeling as sequence and postgenomic data accrue.

### Genomic BLUP

**Figure 9:**
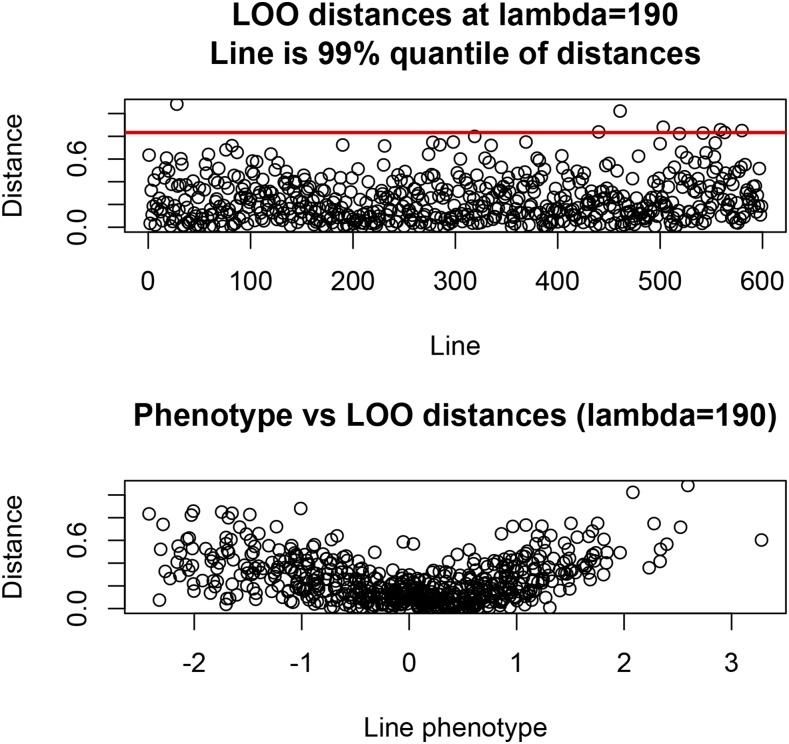
Euclidean distances between genomic BLUP (GBLUP) with all observations and leave-one-out (LOO) GBLUP, by line removed from training in the CV. Bottom panel depicts the association between phenotype left out in training and distances. The regularization parameter used was $\lambda =\frac{\sigma_{e}^{2}}{\sigma_{\beta }^{2}}=190$.

Standard GBLUP, $\hat{g}\left(\lambda \right),$ of genotypic values of the 599 lines $\left({\hat{g}}_{i};\;i=1,2,\ldots ,599\right)$ was computed with λ=190. Subsequently, ${\hat{g}}_{\left[-i\right]}\left(\lambda \right)$ was obtained for each of the 599 lines, *i.e.*, the GBLUP of all lines after removing line *i* in the training process. Euclidean distances between $\hat{g}\left(\lambda \right)$ and ${\hat{g}}_{\left[-i\right]}\left(\lambda \right)$ were calculated as

$$d_{i}\left(\lambda \right)=\sqrt{{\left(\hat{g}\left(\lambda \right)-{\hat{g}}_{\left[-i\right]}\left(\lambda \right)\right)}^{\prime }\left(\hat{g}\left(\lambda \right)-{\hat{g}}_{\left[-i\right]}\left(\lambda \right)\right)};$$ (46)

this metric measures the extent to which removal of line *i* influences model training. The minimum and maximum absolute distances were $3\times 10^{-4}$ and 1.082, respectively, and the coefficient of variation of distances was about 80%. An observation was deemed influential when $d_{i}\left(\lambda \right)≧$0.83, the 99-percentile of the empirical distribution. Figure 9 (top panel) shows a scatter plot of the $d_{i}\left(\lambda \right)$; influential lines (28, 440, 461, 503, 559, and 580) correspond to points on top of the horizontal line. The relationship between the phenotype of the line excluded in the LOO CV is shown in the bottom panel: larger phenotypes tended to be associated with larger distances.

**Figure 10:**
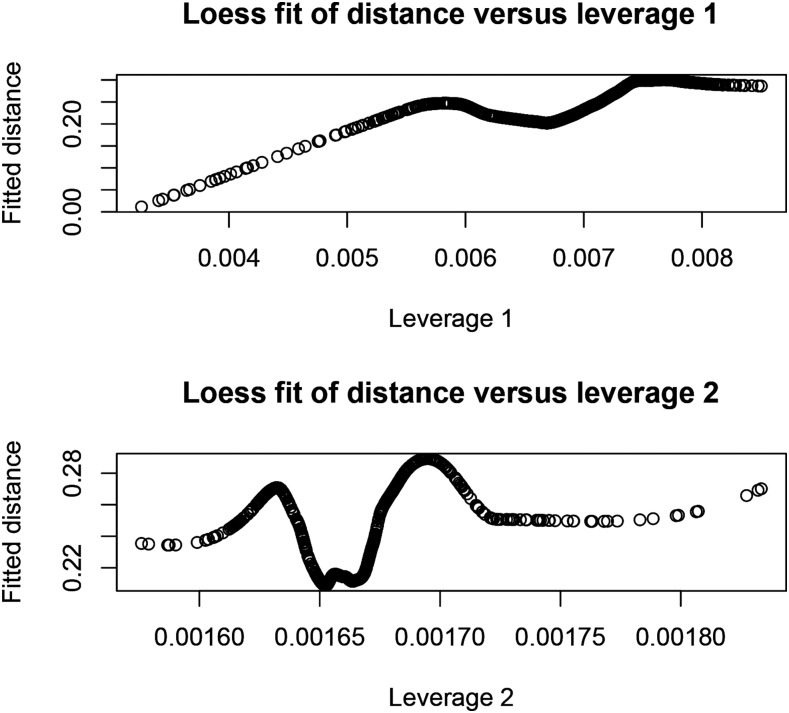
Nonparametric (LOESS) fits of Euclidean distance between GBLUP with all observations and LOO GBLUP on two measures of influence (leverage) a line has on model training. Leverage 1 is the average of absolute values of the regression of all lines on the phenotype of a given line; leverage 2 is the average of such regressions.

Using data from all lines, GBLUP is $\hat{g}=C$ y−1; the influence of phenotype of line *j* =1,2,…,599 on GBLUP of line *i* is element ij of the matrix $\frac{\partial \hat{g}}{\partial y^{\prime }}=C$ ${}^{-1}.$ Observe that

$$C^{-1}=Cov\left(g,y\right)Var^{-1}\left(y\right)={\left(I+G^{-1}\lambda \right)}^{-1}$$ (47)

is as a matrix of n×n regression coefficients; its $i^{th}$ row contains the regression of the genotype of line *i* on the *n* phenotypes. A measure of overall influence (leverage) of line *j* is the average of values (or absolute values) of elements in column *j* of C ${}^{-1}.$ Clearly, leverages depend on relatedness structure and on *λ* but not on phenotypes. Figure 10 depicts plots of LOESS regressions (Cleveland 1979) of Euclidean distance between GBLUP calculated with all lines, and LOO GBLUP on two measures of leverage: the average of absolute values of C ${}^{-1}$ over rows for each line (leverage 1), and the average of elements of C ${}^{-1}$ over rows, by line (leverage 2). LOESS fits (span parameter equal to 0.50) indicated that leverage 1 informs about the impact of removing a specific line in LOO: the larger the leverage 1 of a line, the larger the effect of its removal from the training process.

### RKHS

We built a kernel matrix K with typical element (ranging between 0 and 1)

$$k_{ij}=w_{1}\;\text{exp}\left[-h_{1}\frac{d_{ij}}{\text{max}\left(d_{ij}\right)}\right]+w_{2}\;\text{exp}\left[-h_{2}\frac{d_{ij}}{\text{max}\left(d_{ij}\right)}\right]+w_{3}\;\text{exp}\left[-h_{3}\frac{d_{ij}}{\text{max}\left(d_{ij}\right)}\right];$$ (48)

$d_{ij}={\left(x_{i}-x_{j}\right)}^{\prime }\left(x_{i}-x_{j}\right)$ for i,j=1,2...,599. Here $x_{i}$ is the 1279×1 vector of marker genotypes in line i; $h_{1},$ $h_{2}$, and $h_{3}$ are bandwidth parameters tuned to establish “global,” “regional” and “local” similarities between individuals (as *h* increases, similarity decreases); $w_{1},w_{2}$, and $w_{3}$ are weights assigned to the three sources of similarity, such that $0<w_{i}<1$ and $\sum_{i=1}^{3}w_{i}=1.$ We arbitrarily chose $h_{1}=\frac{1}{2},$ $h_{2}=2$, and $h_{3}=4,$ and $w_{1}=0.5,$ $w_{2}=0.30$, and $w_{3}=0.20$. From K we created three additional kernels by placing $w_{i}=1$ for i=1,2,3, leading to matrices $K_{1},$ $K_{2}$, and $K_{3}.$ Mean off-diagonal elements of the four kernel matrices were 0.73 $\left(K_{1}\right)$, 0.29 $\left(K_{2}\right)$, 0.09 $\left(K_{3}\right)$, and 0.47 (K); these values can be interpreted as correlations between pairs of individuals. Hence, $K_{1}$ and $K_{3}$ produced the highest and lowest degrees of correlation, respectively; complexity of the models increases from kernel 1 to kernel 3 because the fit to the data increases with *h* (de los Campos *et al.* 2009, 2010).

The four no-intercept RKHS models had the basic form

$$y=K\alpha +e;\alpha ∼N\left(0,K^{-1}\sigma_{\alpha }^{2}\right);e∼N\left(0,I\sigma_{e}^{2}\right);Cov\left(\alpha ,e^{\prime }\right)=0,$$ (49)

where K was either as in (48) or $K_{i}=1,2,3.$ Variance components were estimated by maximum likelihood, producing as estimates of $\lambda =\frac{\sigma_{e}^{2}}{\sigma_{\alpha }^{2}}:$ $\lambda_{1}=0.16,$ $\lambda_{2}=0.30$, $\lambda_{3}=0.32$, and $\lambda_{K}=0.21.$ The effective number of parameters was calculated (*e.g.*, kernel 1) as $p_{1}=tr{\left(I+K^{-1}\lambda_{1}\right)}^{-1},$ yielding 224.5, 319.2, and 376.3 for kernel matrices 1, 2, and 3, respectively; for K the effective number of parameters fitted was 330.3. As expected, model complexity increased as the model became more “local.”

We fitted the four RKHS models to all 599 lines, and conducted a LOO CV for each model. In the fitting process, the corresponding regularization parameter was employed; *e.g.*, for $K_{2},$ $\lambda_{2}=0.30$. For each of the models, RKHS predictions of genotypic values were calculated as

$${\hat{g}}_{i}\left(\lambda_{i},h,w\right)={\left(I+K_{i}^{-1}\lambda_{i}\right)}^{-1}y;\;i=1,2,3,K.$$ (50)

The implicit dependence of predictions on bandwidths (h) and weights (w) is indicated in the notation above, but not used hereinafter. In LOO (line *j* left out in the training process), predictions and predictive mean-squared errors are calculated as for LOO GBLUP, that is,

$${\tilde{g}}_{ij}={\left(1-c_{i}^{jj}\right)}^{-1}\left({\hat{g}}_{ij}-c_{i}^{jj}y_{j}\right);\;j=1,2,\ldots ,n,$$ (51)

where $c_{i}^{jj}$ is the $j^{th}$ diagonal element of ${\left(I+K_{i}^{-1}\lambda_{i}\right)}^{-1},$ and

$$PMSE_{i}\left(1\right)=\frac{1}{n}\sum_{j=1}^{n}{\left(y_{j}-{\tilde{g}}_{ij}\right)}^{2}.$$ (52)

**Figure 11:**
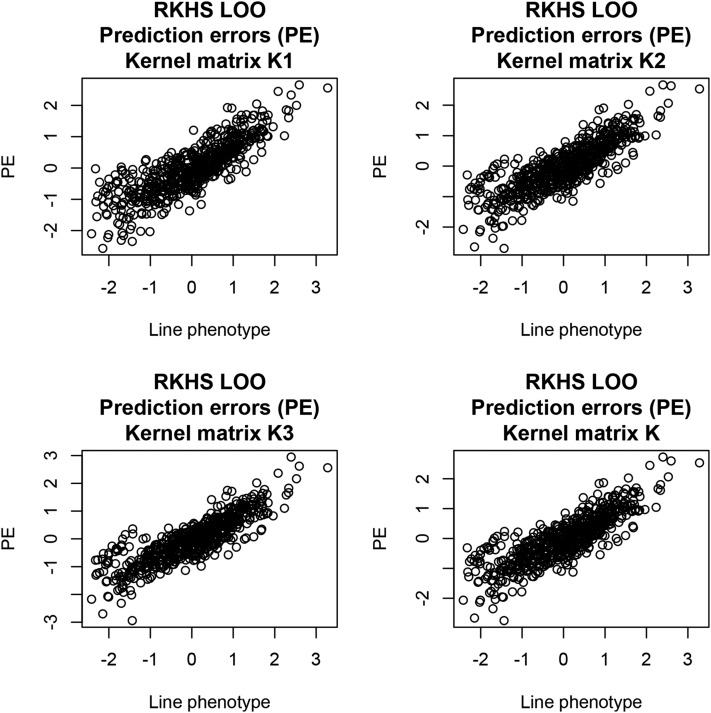
LOO prediction errors (testing set) of four reproducing kernel Hilbert spaces (RKHS) regression models against line phenotypes.

Predictive MSEs were 0.6795 $\left(K_{1}\right),$ 0.6446 $\left(K_{2}\right),$ 0.6555 $\left(K_{3}\right)$, and 0.6439 (K); predictive correlations were 0.566, 0.597, 0.591, and 0.598. Differences between kernels with respect to the criteria used were nil, but the model combining three kernels conveying differing degrees of locality had a marginally smaller MSE and a slightly larger correlation. LOO prediction errors plotted against line phenotypes are shown in Figure 11 for the four kernels used. Prediction errors were larger in absolute value for lines with lowest and highest grain yields, suggesting that the model may benefit by accounting for possibly heterogeneous residual variances. $K_{2}$ and $K_{3}$ captured some substructure in the distribution of fitted residuals. The more global kernel $\left(K_{1}\right),$ arguably capturing mostly additive effects, did not suggest any substructure, which reemerged when the three kernels were combined into K. The preceding exercise illustrates that predictive correlations and PMSE do not fully describe the performance of a prediction machine.

### Bayesian GBLUP with known variance components

Bayesian GBLUP with known variances has a closed form solution: using all data, the posterior distribution is $g|y,\sigma_{e}^{2},\sigma_{g}^{2}∼N\left(\hat{g},C^{-1}\sigma_{e}^{2}\right),$ where $\hat{g}=C^{-1}y$ and $C^{-1}={\left(I+G^{-1}\lambda \right)}^{-1}$. Set $G=XX^{\prime },$ λ=190, and $\sigma_{e}^{2}=0.54;$ X is centered.

This problem was attacked (with Monte Carlo error) by drawing independent samples from the 599-variate normal posterior distribution; no MCMC is needed. Using (34), the importance sampling weights for LOO are

$$w_{i,s}=\frac{p^{-1}\left(y_{i}|g_{i}^{\left(s\right)},0.54\right)}{\sum_{s=1}^{S}p^{-1}\left(y_{i}|g_{i}^{\left(s\right)},0.54\right)};\;\;i=1,2,\ldots ,n;\;\;s=1,2,\ldots ,S,$$ (53)

where $g_{i}^{\left(s\right)}$ is sample *s* for line *i*; $g^{\left(s\right)}$ is drawn from $N\left(\hat{g},C^{-1}0.54\right)$. The importance sampling weight becomes

$$w_{i,s}=\frac{\text{exp}\left[\frac{{\left(y_{i}-g_{i}^{\left(s\right)}\right)}^{2}}{1.08}\right]}{\sum_{s=1}^{S}\text{exp}\left[\frac{{\left(y_{i}-g_{i}^{\left(s\right)}\right)}^{2}}{1.08}\right]}.$$ (54)

Observe that the likelihood that $y_{i}$ confers to $g_{i}^{\left(s\right)}$ is inversely proportional to $w_{i,s}.$ Hence, samples in which the phenotype removed $\left(y_{i}\right)$ confers little likelihood to the $g_{i}$ receive more weight.

**Figure 12:**
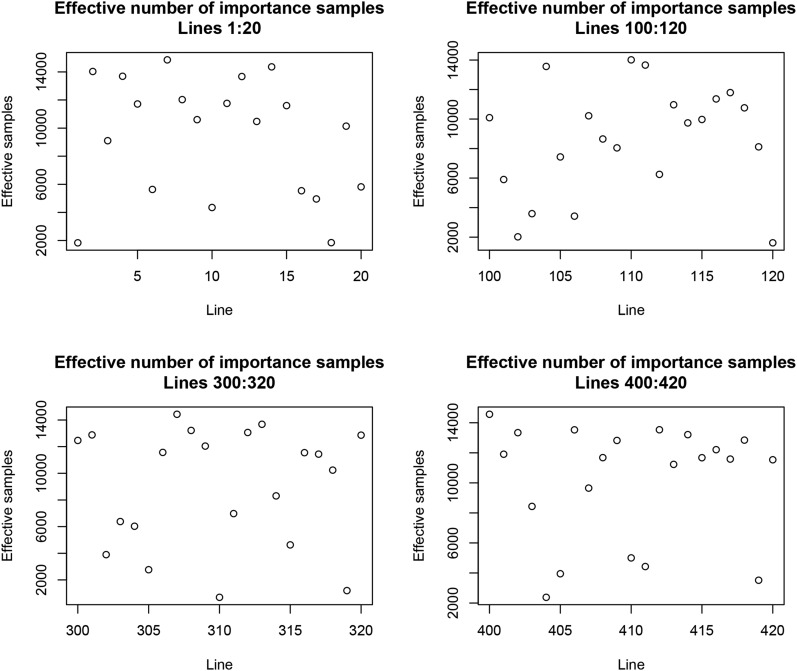
Effective number of importance samples for LOO Bayesian GBLUP (given the variances) for selected lines; 15,000 independent samples were drawn from the posterior distribution of genotypic values.

We took S=15,000 independent samples from $N\left(\hat{g},C^{-1}0.54\right).$ The effective number of weights per line, calculated with (38) ranged from 76.4 to 14,983.5; the median (mean) weight was 10,991.0 (9789.2), and the first and third quartiles of the distribution were 6826.1 and 14,983.5, respectively. On average, 1.54 independent samples were required for drawing an effectively independent LOO posterior sample. Figure 12 illustrates the variability among some arbitrarily selected lines of the mean number of importance weights. A phenotype having a small mean importance weight would be “surprising” with respect to the model. However, there are theoretical and numerical issues with the weights used here, a point retaken in the discussion.

**Figure 13:**
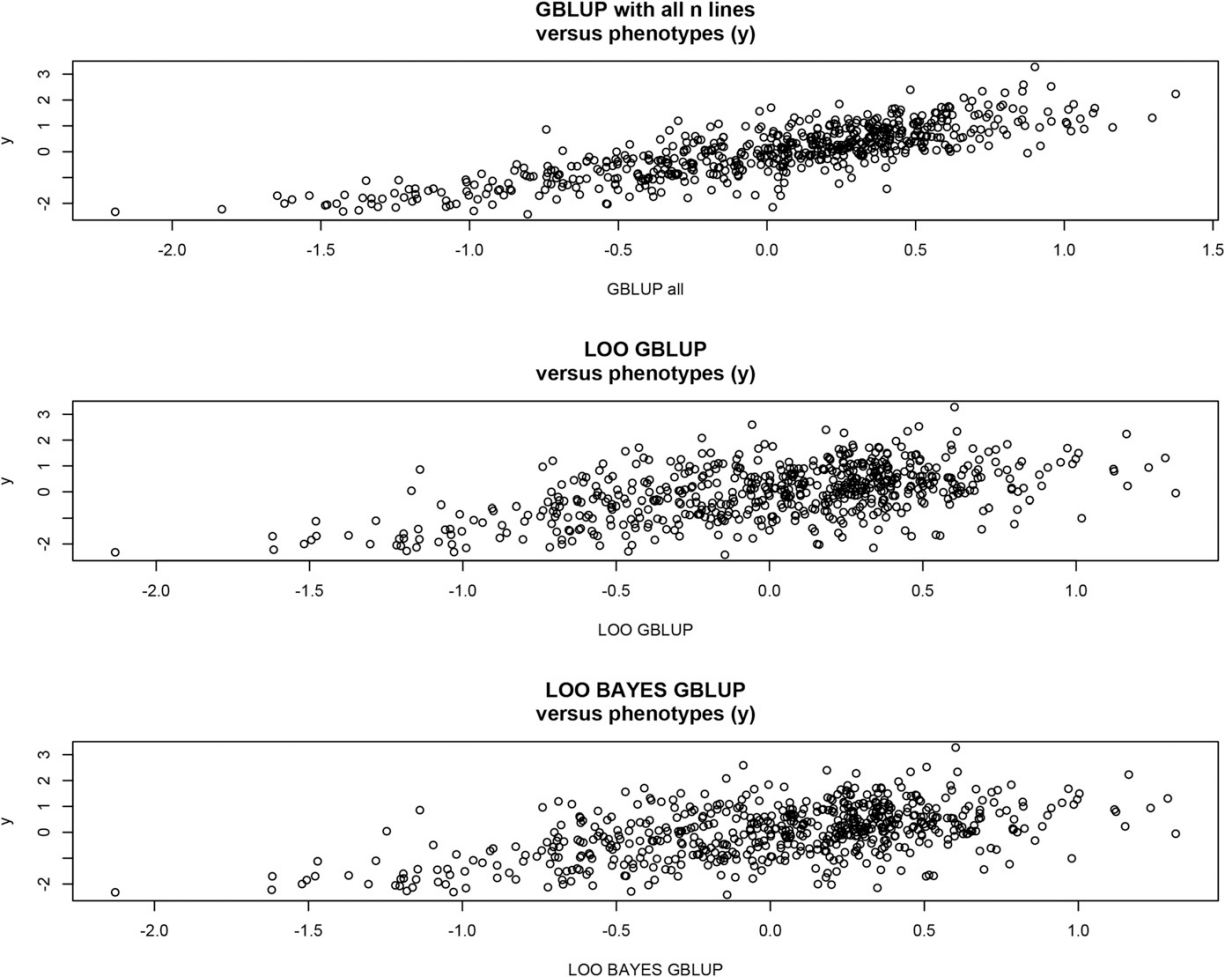
Associations between phenotypes and predictions (GBLUP with all n=599 lines used in training; LOO GBLUP, leave-one-out genomic BLUP; LOO BAYES GBLUP, direct sampling from the posterior distribution of genotypic values of followed by importance sampling to obtained the LOO predictions.

Figure 13 shows that GBLUP using the entire sample of size *n* fitted closely. Correlations between predictors were 0.91 in all cases. Mean-squared errors were 0.40 (GBLUP, entire sample), 0.73 (Bayes LOO), and 0.73 (LOO GBLUP). The correlation between predictions and phenotypes was 0.81 for GBLUP (entire sample), 0.52 for LOO GBLUP, and 0.51 for Bayes LOO.

The importance sampling scheme worked well. Ionides (2008) suggested a modification of the importance ratio assigned to sample *s* and data point i, $r_{i,s}=p^{-1}\left(y_{i}|\beta^{\left(s\right)},\sigma_{e}^{2\left(s\right)}\right)$, as follows

$${r\prime }_{i,s}=\text{min}\left(r_{i,s},\sqrt{S}{\bar{r}}_{i}\right),$$ (55)

where ${\bar{r}}_{i}$ is the average (over samples) importance sample ratio for line *i* in the context of the wheat data set. Ionides (2008) argued that truncation of large importance sampling weights (TIS) would be less sensitive with respect to the importance sampling density function used (the posterior distribution using all data points in our case) than IS. We converted the IS weights into normalized TIS weights using rule (55) applied to the normalized IS weights. As mentioned earlier, the effective number of normalized IS weights ranged between 76.4 and 14983.5; for normalized TIS weights, the effective number spanned from 691.9 to 13,970. TIS produced more stable weights than standard IS. However, truncation of weights introduces a bias, which may affect predictive performance adversely (Vehtari *et al.* 2016). However, it may be that TIS made the weights “too homogeneous,” thus creating a bias toward the posterior distribution obtained with all data points. If all weights were equal to a constant, IS or TIS would retrieve the full posterior distribution.

## Discussion

Cross-validation (CV) has become an important tool for calibrating prediction machines in genome-enabled prediction (Meuwissen *et al.* 2001), and is often preferred over resampling methods such as bootstrapping. It gives a means for comparing and calibrating methods and training sets (*e.g.*, Isidro *et al.* 2015). Typically, CV requires dividing data into training and testing folds, and the models must be run many times. An extreme form of CV is LOO; here if the sample has size n, each observation is removed in the training process, and labeled as a testing set of size 1. Hence, *n* different models must be run to complete a LOO CV.

Our paper presented statistical methodology aimed at enabling extensive CV in genome-enabled prediction using a suite of methods. These were OLS, BLUP on markers, GBLUP, RKHS, and Bayesian procedures. Formulae were derived that enable arriving at the predictions that would be obtained if one or more cases were to be excluded from the training process and declared as members of the testing set. In the cases of OLS, BLUP, GBLUP, and RKHS, and assuming that the ratio of variance components do not change appreciably from those that apply to the entire sample, the formulae are exact.

The deterministic formulae can also be applied in a multiple-kernel or multiple-random factors setting, given the variance components. For example, consider the bikernel RKHS regression (*e.g.*, de los Campos *et al.* 2010; Tusell *et al.* 2014)

$$\begin{array}{c} y=g_{P}+g_{M}+e\\ =K_{P}\alpha_{P}+K_{M}\alpha_{M}+e. \end{array}$$ (56)

Here, $g_{P}=$ $K_{P}\alpha_{P}$ is genetic signal captured by pedigree, and $g_{M}=K_{M}\alpha_{M}$ is genetic signal captured by markers; $\alpha_{P}$ and $\alpha_{M}$ are unknown and independently distributed RKHS regression coefficients, and $K_{P}$ and $K_{M}$ are positive-definite similarity matrices. Suppose that $K_{P}=A$, *i.e.*, the numerator relationship matrix based on the assumption of additive inheritance, and that $K_{M}$ is a Gaussian kernel such as those employed earlier in the paper. The standard assumption for the RKHS regression coefficients is

$$\left[\begin{array}{c} \alpha_{P}\\ \alpha_{M} \end{array}\right]∼N\left(\left[\begin{array}{c} 0\\ 0 \end{array}\right],\left[\begin{array}{cc} A^{-1}\sigma_{\alpha_{A}}^{2} & 0\\ 0 & K_{M}^{-1}\sigma_{\alpha_{M}}^{2} \end{array}\right]\right)$$ (57)

where $\sigma_{\alpha_{A}}^{2}$ and $\sigma_{\alpha_{M}}^{2}$ are variance components associated with kernels A and $K_{M},$ respectively. Hence, $g_{P}∼N\left(0,A\sigma_{\alpha_{A}}^{2}\right)$ and $g_{M}∼N\left(0,K_{M}\sigma_{\alpha_{M}}^{2}\right).$ The BLUP of $g_{P}$ and $g_{M}$ can be found by solving the linear system

$$\left[\begin{array}{cc} I+A^{-1}\lambda_{A} & I\\ I & I+K_{M}^{-1}\lambda_{M} \end{array}\right]\left[\begin{array}{c} {\hat{g}}_{P}\\ {\hat{g}}_{M} \end{array}\right]=\left[\begin{array}{c} y\\ y \end{array}\right],$$ (58)

where $\lambda_{A}=\frac{\sigma_{e}^{2}}{\sigma_{\alpha_{A}}^{2}}$ and $\lambda_{M}=\frac{\sigma_{e}^{2}}{\sigma_{\alpha_{M}}^{2}}$. Hence, the solutions satisfy

$${\hat{g}}_{P}={\left(I+A^{-1}\lambda_{A}\right)}^{-1}\left(y-{\hat{g}}_{M}\right)=C_{A}^{-1}\left(y-{\hat{g}}_{M}\right),$$ (59)

$${\hat{g}}_{M}={\left(I+K_{M}^{-1}\lambda_{M}\right)}^{-1}\left(y-{\hat{g}}_{P}\right)=C_{M}^{-1}\left(y-{\hat{g}}_{P}\right).$$ (60)

Applying the logic leading to (102) for the LOO situation, the preceding equations can be written as

$${\tilde{g}}_{i,P}=\frac{1}{1-c_{A}^{ii}}\left[{\hat{g}}_{i,P}-c_{A}^{ii}\left(y_{i}-{\hat{g}}_{i,M}\right)\right];$$ (61)

$${\tilde{g}}_{i,M}=\frac{1}{1-c_{M}^{ii}}\left[{\hat{g}}_{i,M}-c_{M}^{ii}\left(y_{i}-{\hat{g}}_{i,P}\right)\right],$$ (62)

where $c_{A}^{ii}\left(c_{M}^{ii}\right)$ are the $i^{th}$ diagonal elements of $C_{A}^{-1}\left(C_{M}^{-1}\right),$ respectively; ${\hat{g}}_{i,P}$ and ${\hat{g}}_{i,M}$ are the corresponding solutions to the system of equations (58). The prediction of the left-out phenotype is ${\tilde{y}}_{i}={\tilde{g}}_{i,P}+{\tilde{g}}_{i,M}.$

The situation is different for Bayesian models solved by sampling methods such as MCMC. Typically, there is no closed form solution, except in some stylized situations, so sampling must be used. The Bayesian model must be run with the entire data set and posterior samples weighted, *e.g.*, via importance sampling, to convert realizations into draws pertaining to the posterior distribution that would result from using just the CV training set. Unfortunately, importance weights can be extremely variable. In (34), it can be seen that weights are proportional to the reciprocal of likelihoods evaluated at the posterior samples, so if a data point (or a vector of data points) “left out” confers a tiny likelihood to the realized value of the parameter sampled, then the sample is assigned a very large weight; on the other hand, if the likelihood is large, the weight is small. This phenomenon produces a large variance among weights, which we corroborated empirically. Another view at the issue at stake is as follows: the importance weight is $w_{i}\left(\beta \right)=\frac{p\left(\beta |y_{-i},H\right)}{p\left(\beta |y,H\right)};$ hence, if the posterior obtained with all data points has much thinner tails than the posterior density constructed by excluding one or more cases, the weights can “blow up.”

The preceding problem would be exacerbated by including more than one observation in the testing set. Vehtari *et al.* (2016) examined seven data sets, and used “brute force” LOO MSE as a gold standard to examine the performance of various forms of importance sampling. TIS gave a better performance than standard importance sampling (IS) weights in two out of seven comparisons, with the standard method being better in three of the data sets; there were two ties. Vehtari *et al.* (2016) suggested another method called Pareto smoothed importance sampling (PSIS) that was better than IS in four of the data sets (two ties), and better than TIS in three of the analyses (two ties). Calculation of PSIS is involved, requiring several steps in our context: (a) compute IS ratios; (b) for each of the 599 lines, fit a generalized Pareto distribution to the 20% largest values found in (a); (c) replace the largest *M* IS ratios by expected values of the order statistics of the fitted generalized Pareto distribution, where M=0.20×S; (d) for each line, truncate the new ratios. Clearly, this procedure does not lend itself to large scale genomic data, and the results of Vehtari *et al.* (2016), obtained with small data sets and simple models, are not conclusive enough. Additional research is needed to examine whether TIS, IS, or PSIS are better for genome-enabled prediction.

It is known (*e.g.*, Henderson 1973, 1984; Searle 1974) that the best predictor, that is, the function of the data with the smallest squared prediction error (MSE) under conceptual repeated sampling (*i.e.*, infinite number of repetitions over the joint distribution of predictands and predictors) is E(g|y,parameters). This property requires knowledge of the form of the joint distribution, and of its parameters. In the setting of the case study, under multivariate normality, and with a zero-mean model and known variance components, GBLUP is the best predictor. However, in CV, the property outlined above does not hold. One reason is that the data set represents a single realization of the conceptual scheme. Another reason is that incidence and similarity matrices change at random in CV, plus parameters are estimated from the data at hand. For example, if datum *i* is removed from the analysis, the training model genomic relationship matrix becomes $G_{\left[-i,-i\right]},$ whereas, if observation *j* is removed, the matrix used is $G_{\left[-j,-j\right]}.$ Further, the entire data set is used in the CV, so yet-to-be observed data points appear in the training process at some point. The setting of best prediction requires that the structure of the data remains constant over repeated sampling, with the only items changing being the realized values of the data (y), and of the unobserved genotypic values (g). The CV setting differs from the idealized scheme, and expectations based on theory may not always provide the best effective guidance in predictive inference.

In conclusion, CV appears to be the best gauge for calibrating prediction machines. Results presented here provide the basis for conducting extensive cross-validation from results of a single run with all data. Future research should evaluate importance sampling schemes for more complex Bayesian models, *e.g.*, those using thick-tailed processes or mixtures as prior distributions. An important challenge is to make the procedures developed here computationally cost-effective, so that software for routine use can be developed.

## Appendix A: Prediction with Least-Squares

Following Seber and Lee (2003), consider out-of-sample prediction, and suppose that the distribution of residuals in left-out data (testing set) of size $n_{test}$ is as in a training set of size *n*, with the training phenotypes represented as y; residuals in training and testing sets are assumed to be mutually independent. In genome-enabled prediction, (1) is merely an instrumental model that may bear little resemblance with the state of nature, so we let the true distribution be $y_{test}∼N\left(\mu ,I\sigma_{e}^{2}\right),$ allowing for the almost certain possibility that $\mu \neq X_{test}\beta$, where $X_{test}$ is the marker matrix in the testing set. In quantitative genetics, μ is an unknown function of quantitative trait locus (QTL) genotypes and of their effects; the latter may not be additive, and dominance or epistasis may enter into the picture without contributing detectable variance.

Model training yields $\hat{\beta }$, and the point predictor of $y_{test}$ is $X_{test}\hat{\beta }$. The mean squared error of prediction (PMSE), conditionally on training data (y,X) and on $X_{test}$ but averaged with respect of all possible testing sets of size, $n_{test}$, is

$$E\left(\text{PMSE}|y,X,X_{test}\right)=\frac{1}{n_{test}}E_{y_{test}|y,X,X_{test}}\left[{\left(y_{test}-X_{test}\hat{\beta }\right)}^{\prime }\left(y_{test}-X_{test}\hat{\beta }\right)\right].$$ (63)

Define the conditional prediction bias as

$$\hat{\delta }=E_{y_{test}|y,X,X_{test}}\left(y_{test}-X_{test}\hat{\beta }|y\right)=\mu -X_{test}\hat{\beta }.$$ (64)

Standard results on expectation of quadratic forms applied to (63) produce

$$\begin{array}{c} E\left(\text{PMSE}|y,X,X_{test}\right)=\frac{1}{n_{test}}\left\{{\left(\mu -X_{test}\hat{\beta }\right)}^{\prime }\left(\mu -X_{test}\hat{\beta }\right)+tr\left[Var\left(y_{test}\right)\right]\right\}\\ =\frac{1}{n_{test}}\left[\hat{\delta^{\prime }}\hat{\delta }+n_{test}\sigma_{e}^{2}\right]. \end{array}$$ (65)

Unconditionally, that is, by averaging over all possible sets of training (testing) data of size *n* $\left(n_{test}\right)$ with marker configuration X $\left(X_{test}\right)$

$$E\left(\text{PMSE}|X,X_{test}\right)=E_{y|X,X_{test}}\left[E\left(\text{PMSE}|y,X,X_{test}\right)\right]=\frac{1}{n_{test}}\left[E_{{}_{y|X,X_{test}}}\left({\hat{\delta }}^{\prime }\hat{\delta }\right)+n_{test}\sigma_{e}^{2}\right],$$ (66)

where

$$\begin{array}{c} E_{{}_{y|X,X_{test}}}\left({\hat{\delta }}^{\prime }\hat{\delta }\right)={\left[\mu -X_{test}E\left(\hat{\beta }\right)\right]}^{\prime }\left[\mu -X_{test}E\left(\hat{\beta }\right)\right]+\sigma_{e}^{2}tr\left[X_{test}{\left(X^{\prime }X\right)}^{-1}{X^{\prime }}_{test}\right]\\ =\delta^{\prime }\delta +\sigma_{e}^{2}tr\left[X_{test}{\left(X^{\prime }X\right)}^{-1}{X^{\prime }}_{test}\right]. \end{array}$$ (67)

Here, the expected prediction bias is

$$\delta =\mu -X_{test}E\left(\hat{\beta }\right)=\left[I-X_{test}{\left(X^{\prime }X\right)}^{-1}X^{\prime }\right]\mu ,$$ (68)

after assuming for convenience of representation that $E\left(y_{test}\right)=E\left(y\right)=\mu ,$ *i.e.*, that testing and training sets have the same size and unknown true mean μ; note that $\frac{\partial X_{test}\hat{\beta }}{\partial y^{\prime }}=X_{test}{\left(X^{\prime }X\right)}^{-1}X^{\prime }$ is a matrix of “influences” describing how variation in training phenotypes affects predictions. Using (68) in (67), and this in (66), the expected prediction mean-squared error averaged over an infinite number of training and testing sets, but with fixed marker genotype matrices is

$$E\left(\text{PMSE}|X,X_{test}\right)=\frac{1}{n_{test}}\left\{\delta^{\prime }\delta +\sigma_{e}^{2}tr\left[X_{test}{\left(X^{\prime }X\right)}^{-1}{X^{\prime }}_{test}\right]+n_{test}\sigma_{e}^{2}\right\}$$ (69)

If $X_{test}=X,$ that is, in the special case where the same genotypes appear in the training and testing sets, $X_{test}{\left(X^{\prime }X\right)}^{-1}{X^{\prime }}_{test}=H=\left\{h_{ij}\right\}$ and $tr\left(X_{test}{\left(X^{\prime }X\right)}^{-1}{X^{\prime }}_{test}\right)=p,$ so the preceding equation becomes

$$E\left(\text{PMSE}|X\right)=\frac{1}{n_{test}}\sum_{i=1}^{n}\delta_{i}^{2}+\left(1+\frac{p}{n_{test}}\right)\sigma_{e}^{2},$$ (70)

with

$$\delta =\left\{\delta_{i}\right\}=\left[I-X{\left(X^{\prime }X\right)}^{-1}X^{\prime }\right]\mu =\left(I-H\right)\mu ,$$ (71)

and typical element $\delta_{i}=\left(1-\sum_{j=1}^{n}h_{ij}\right)\mu_{i}$. Expression (70) shows that the uncertainty of prediction, as measured by variance (second term) increases with *p* (model complexity) and decreases with $n_{test}$ (equal to *n* here). The impact of increasing complexity on bias is difficult to discuss in the absence of knowledge of QTL and their effects. When p→n, the training data set fits better and better and the model increasingly copies both signal and error: predictions become increasingly poorer.

Note that

$$Var\left[\left(y-X\hat{\beta }\right)\right]=MM\sigma_{e}^{2}=\left(I-H\right)\sigma_{e}^{2},$$ (72)

where $M=I-X{\left(X^{\prime }X\right)}^{-1}X^{\prime }=I-H$. Applying this result in (8)

$$E_{y|X}\left[\text{PMSE}\left(1\right)\right]=\frac{1}{n}\left[\delta^{\prime }D\delta +\sigma_{e}^{2}tr\left(DM\right)\right],$$ (73)

where $\frac{1}{n}\left(\delta^{\prime }D\delta \right)$ is the average squared prediction bias, and $\frac{\sigma_{e}^{2}}{n}tr\left[DM\right]$ is the average prediction error variance. Note that

$$\delta^{\prime }D\delta =\sum_{i=1}^{n}\frac{\delta_{i}^{2}}{{\left(1-h_{ii}\right)}^{2}},$$ (74)

and that

$$tr\left(DM\right)=tr\left(D-DH\right)=\sum_{i=1}^{n}\left(\frac{1}{{\left(1-h_{ii}\right)}^{2}}-\frac{h_{ii}}{{\left(1-h_{ii}\right)}^{2}}\right)=\sum_{i=1}^{n}\left[\frac{1-h_{ii}}{{\left(1-h_{ii}\right)}^{2}}\right].$$ (75)

Employing (74) and (75) in (73),

$$\begin{array}{c} E_{y|X}\left[\text{PMSE}\left(1\right)\right]=\frac{1}{n}\left\{\sum_{i=1}^{n}[\frac{\delta_{i}^{2}}{{\left(1-h_{ii}\right)}^{2}}+\sigma_{e}^{2}\sum_{i=1}^{n}\left(\frac{1-h_{ii}}{{\left(1-h_{ii}\right)}^{2}}\right)]\right\}\\ =\frac{1}{n}\sum_{i=1}^{n}\left[\frac{\delta_{i}^{2}+\sigma_{e}^{2}\left(1-h_{ii}\right)}{{\left(1-h_{ii}\right)}^{2}}\right]. \end{array}$$ (76)

Examine the difference $\left(\Delta_{MSE}\right)$ in expected prediction mean-squared error between the LOO procedure, as given in (76), and that from the “distinct testing set” layout given in (70), and set $n=n_{test}$. One obtains

$$\Delta_{MSE}=\frac{1}{n}\sum_{i=1}^{n}\left[\frac{\delta_{i}^{2}+\sigma_{e}^{2}\left(1-h_{ii}\right)}{{\left(1-h_{ii}\right)}^{2}}\right]-\frac{1}{n}\left[\sum_{i=1}^{n}\delta_{i}^{2}+\left(n+p\right)\sigma_{e}^{2}\right].$$ (77)

Since $p=tr\left(X{\left(X^{\prime }X\right)}^{-1}X^{\prime }\right)=tr\left(H\right)=\sum_{i=1}^{n}h_{ii},$

$$\begin{array}{c} \Delta_{MSE}=\frac{1}{n}\left\{\sum_{i=1}^{n}\left[\frac{\delta_{i}^{2}+\sigma_{e}^{2}\left(1-h_{ii}\right)}{{\left(1-h_{ii}\right)}^{2}}\right]-\sum_{i=1}^{n}\left[\delta_{i}^{2}+\left(1+h_{ii}\right)\sigma_{e}^{2}\right]\right\}\\ =\frac{1}{n}\left\{\sigma_{e}^{2}{\sum_{i=1}^{n}\frac{h_{ii}^{2}}{1-h_{ii}}}_{}^{}+\sum_{i=1}^{n}\frac{\delta_{i}^{2}h_{ii}\left(2-h_{ii}\right)}{{\left(1-h_{ii}\right)}^{2}}\right\}. \end{array}$$ (78)

The $h_{ii}$ are bounded between 0 and 1, so the two terms in (78) are positive.

### Appendix B: Matrix Algebra Result

Early derivations of the mixed model equations used for computing best linear unbiased estimation of fixed effects, and BLUP of random effects in mixed linear models used by Henderson’s (*e.g.*, Henderson *et al.* 1959; Henderson 1975, 1984) made use of the Sherman-Morrison-Woodbury formula (Seber and Lee 2003). Moore-Penrose generalizations are in Deng (2011).

Assuming that the required inverses stated below exist, the following result holds

$${\left(A+UBV\right)}^{-1}=A^{-1}-A^{-1}UB{\left(B+BVA^{-1}UB\right)}^{-1}BVA^{-1}.$$ (79)

A special case is when B=I, U=±u, $V=v^{\prime },$ where u and $v^{\prime }$ denote column and row vectors; here

$${\left(A+uv^{\prime }\right)}^{-1}=A^{-1}-\frac{A^{-1}uv^{\prime }A^{-1}}{1+v^{\prime }A^{-1}u},$$ (80)

$${\left(A-uv^{\prime }\right)}^{-1}=A^{-1}+\frac{A^{-1}uv^{\prime }A^{-1}}{1-v^{\prime }A^{-1}u}.$$ (81)

### Appendix C: LOO Ordinary Least-Squares

In (4), we have

$$\begin{array}{c} {\hat{\beta }}_{\left[-i\right]}={\left({X^{\prime }}_{\left[-i\right]}X_{\left[-i\right]}\right)}^{-1}{X^{\prime }}_{\left[-i\right]}y_{\left[-i\right]}\\ ={\left(X^{\prime }X-x_{i}{x^{\prime }}_{i}\right)}^{-1}\left(X^{\prime }y-x_{i}y_{i}\right)\\ =\left[{\left(X^{\prime }X\right)}^{-1}+\frac{{\left(X^{\prime }X\right)}^{-1}x_{i}{x^{\prime }}_{i}{\left(X^{\prime }X\right)}^{-1}}{1-{x^{\prime }}_{i}{\left(X^{\prime }X\right)}^{-1}x_{i}}\right]\left(X\prime y-x_{i}y_{i}\right). \end{array}$$ (82)

Let the n×n regression “hat matrix” be $H=X{\left(X^{\prime }X\right)}^{-1}X^{\prime }$, with diagonal elements $h_{ii}=x_{i}^{\prime }{\left(X^{\prime }X\right)}^{-1}x_{i};$ i=1,2,…,n. Hence, (4) can be written as

$$\begin{array}{c} {\hat{\beta }}_{\left[-i\right]}={\left(X^{\prime }X\right)}^{-1}X^{\prime }y-{\left(X^{\prime }X\right)}^{-1}x_{i}y_{i}\\ +\frac{{\left(X^{\prime }X\right)}^{-1}x_{i}{x^{\prime }}_{i}{\left(X^{\prime }X\right)}^{-1}X^{\prime }y}{1-h_{ii}}-\frac{{\left(X^{\prime }X\right)}^{-1}x_{i}{x^{\prime }}_{i}{\left(X^{\prime }X\right)}^{-1}x_{i}y_{i}}{1-h_{ii}}\\ =\hat{\beta }-\frac{\left(1-h_{ii}\right){\left(X^{\prime }X\right)}^{-1}x_{i}y_{i}-{\left(X^{\prime }X\right)}^{-1}x_{i}{x^{\prime }}_{i}\hat{\beta }+h_{ii}{\left(X^{\prime }X\right)}_{i}^{-1}xy_{i}}{1-h_{ii}}\\ =\hat{\beta }-\frac{{\left(X^{\prime }X\right)}^{-1}x_{i}\left(y_{i}-{x^{\prime }}_{i}\hat{\beta }\right)}{1-h_{ii}}=\hat{\beta }-\frac{{\left(X^{\prime }X\right)}^{-1}x_{i}{\hat{e}}_{i}}{1-h_{ii}}. \end{array}$$ (83)

Using (83), the error of fitting phenotype $y_{i}$ is then

$$\begin{array}{c} y_{i}-{x^{\prime }}_{i}{\hat{\beta }}_{\left[-i\right]}=y_{i}-\left[{x^{\prime }}_{i}\hat{\beta }-\frac{{x^{\prime }}_{i}{\left(X^{\prime }X\right)}^{-1}x_{i}\left(y_{i}-{x^{\prime }}_{i}\hat{\beta }\right)}{1-h_{ii}}\right]=y_{i}-{x^{\prime }}_{i}\hat{\beta }+\frac{h_{ii}\left(y_{i}-{x^{\prime }}_{i}\hat{\beta }\right)}{1-h_{ii}}\\ =\frac{y_{i}-{x^{\prime }}_{i}\hat{\beta }}{1-h_{ii}};\;\;i=1,2,\ldots ,n. \end{array}$$ (84)

### Appendix D: Leave-d-Out Ordinary Least-Squares

If *d* cases are removed from the training data set, $X_{\left[-d\right]}^{\prime }X_{\left[-d\right]}=X^{\prime }X-X_{\left[d\right]}^{\prime }X_{\left[d\right]}.$ In (79), put $A=X^{\prime }X,$ $U=X_{\left[d\right]}^{\prime },$ B=−I, and $V=X_{\left[d\right]}$, to obtain the coefficient matrix

$${\left(X^{\prime }X-X_{\left[d\right]}^{\prime }X_{\left[d\right]}\right)}^{-1}={\left(X^{\prime }X\right)}^{-1}+{\left(X^{\prime }X\right)}^{-1}X_{\left[d\right]}^{\prime }{\left[I-X_{\left[d\right]}{\left(X^{\prime }X\right)}^{-1}X_{\left[d\right]}^{\prime }\right]}^{-1}X_{\left[d\right]}{\left(X^{\prime }X\right)}^{-1},$$ (85)

for *d* being one of the $D=\left(\begin{array}{c} n\\ d \end{array}\right)$ possible d−tuplets. For example, if n=1000, and d=100 or 500, then $D=6.39\times 10^{139}$ and $2.71\times 10^{299},$ respectively. Clearly, it is not feasible to consider all possible configurations of training and testing sets. Note that ${\left[I-X_{\left[d\right]}{\left(X^{\prime }X\right)}^{-1}X_{\left[d\right]}^{\prime }\right]}^{-1}$ will not exist for all combinations of *n* and *d*. For the leave-*d*-out least-squares estimator given to exist, it is needed that n−d≥p.

The right-hand side vector in least-squares takes the form

$$X_{\left[-d,\right]}^{\prime }y_{\left[-d\right]}=\sum_{i=1}^{n}x_{i}y_{i}-\sum_{j\in d}x_{j}y_{d}=X^{\prime }y-X_{\left[d\right]}^{\prime }y_{\left[d\right]}.$$ (86)

Let $\;H_{d}=X_{\left[d\right]}{\left(X^{\prime }X\right)}^{-1}X_{\left[d\right]}^{\prime }$ be the d×d part of the hat matrix H. The OLS estimator, ${\hat{\beta }}_{\left[-d\right]}={\left(X_{\left[-d,\right]}^{\prime }X_{\left[-d,\right]}\right)}^{-1}X_{\left[-d,\right]}^{\prime }y_{\left[-d\right]}$, can be written as

$$\begin{array}{c} {\hat{\beta }}_{\left[-d\right]}=\left\{{\left(X^{\prime }X\right)}^{-1}+{\left(X^{\prime }X\right)}^{-1}{X^{\prime }}_{\left[d\right]}{\left[I-H_{d}\right]}^{-1}X_{\left[d\right]}{\left(X^{\prime }X\right)}^{-1}\right\}\left(X^{\prime }y-{X^{\prime }}_{\left[d\right]}y_{\left[d\right]}\right)\\ =\hat{\beta }-{\left(X^{\prime }X\right)}^{-1}{X^{\prime }}_{\left[d\right]}y_{\left[d\right]}\\ +{\left(X^{\prime }X\right)}^{-1}{X^{\prime }}_{\left[d\right]}{\left[I-H_{d}\right]}^{-1}X_{\left[d\right]}{\left(X^{\prime }X\right)}^{-1}X^{\prime }y\\ \;\;\;-{\left(X^{\prime }X\right)}^{-1}{X^{\prime }}_{\left[d\right]}{\left[I-H_{d}\right]}^{-1}X_{\left[d\right]}{\left(X^{\prime }X\right)}^{-1}{X^{\prime }}_{\left[d\right]}y_{\left[d\right]}. \end{array}$$ (87)

Further,

$$\begin{array}{c} {\hat{\beta }}_{\left[-d\right]}=\hat{\beta }-{\left(X^{\prime }X\right)}^{-1}{X^{\prime }}_{\left[d\right]}y_{\left[d\right]}\\ \;\;\;+{\left(X^{\prime }X\right)}^{-1}{X^{\prime }}_{\left[d\right]}{\left(I-H_{d}\right)}^{-1}X_{\left[d\right]}\hat{\beta }-{\left(X^{\prime }X\right)}_{\left[d\right]}^{-1}{X^{\prime }}_{\left[d\right]}{\left(I-H_{d}\right)}^{-1}H_{d}y_{\left[d\right]}\\ =\hat{\beta }-{\left(X^{\prime }X\right)}^{-1}{X^{\prime }}_{\left[d\right]}{\left(I-H_{d}\right)}^{-1}\left[\left(I-H_{d}\right)y_{\left[d\right]}-X_{\left[d\right]}\hat{\beta }+H_{d}y_{\left[d\right]}\right]\\ =\hat{\beta }-{\left(X^{\prime }X\right)}^{-1}{X^{\prime }}_{\left[d\right]}{\left(I-H_{d}\right)}^{-1}\left(y_{\left[d\right]}-X_{\left[d\right]}\hat{\beta }\right)\\ =\hat{\beta }-{\left(X^{\prime }X\right)}^{-1}{X^{\prime }}_{\left[d\right]}{\left(I-H_{d}\right)}^{-1}{\hat{e}}_{\left[d\right]}. \end{array}$$ (88)

### Appendix E: LOO and Leave-d-Out BLUP of Markers

As indicated in (15), finding BLUP of markers requires computing the coefficient matrix in the corresponding equations, and the vector of right hand sides. Following the reasoning used for least-squares

$$X^{\prime }X+I\lambda =\sum_{i=1}^{n}x_{i}x_{i}^{\prime }+I\lambda$$ (89)

If observation $\left(y_{i},x_{i}^{\prime }\right)$ is to be predicted in a LOO CV, $X_{\left[-i,\right]}^{\prime }X_{\left[-i,\right]}+I\lambda =X^{\prime }X+I\lambda -x_{i}x_{i}^{\prime }$ and $X_{\left[-i,\right]}^{\prime }y_{\left[-i,\right]}=X^{\prime }y-x_{i}y_{i}$, so

$$\begin{array}{c} \beta_{\left[-i\right]}^{r}={\left(X_{\left[-i,\right]}^{\prime }X_{\left[-i,\right]}+I\lambda \right)}^{-1}X_{\left[-i,\right]}^{\prime }y_{\left[-i,\right]}\\ ={\left(X^{\prime }X+I\lambda -x_{i}x_{i}^{\prime }\right)}^{-1}\left(X^{\prime }y-x_{i}y_{i}\right)\\ =\left[{\left(X^{\prime }X+I\lambda \right)}^{-1}+\frac{{\left(X^{\prime }X+I\lambda \right)}^{-1}x_{i}x_{i}^{\prime }{\left(X^{\prime }X+I\lambda \right)}^{-1}}{1-h_{ii}^{r}}\right]\left(X^{\prime }y-x_{i}y_{i}\right), \end{array}$$

where $h_{ii}^{r}=x_{i}^{\prime }{\left(X^{\prime }X+I\lambda \right)}^{-1}x_{i}.$ Letting $C^{-1}={\left(X^{\prime }X+I\lambda \right)}^{-1},$ the preceding becomes

$$\begin{array}{c} \beta_{\left[-i\right]}^{r}=C^{-1}X^{\prime }y-C^{-1}x_{i}y_{i}\\ \;\;+\frac{C^{-1}x_{i}x_{i}^{\prime }C^{-1}X^{\prime }y}{1-h_{ii}^{r}}-\frac{C^{-1}x_{i}x_{i}^{\prime }C^{-1}x_{i}y_{i}}{1-h_{ii}^{r}}\\ =\beta^{r}-C^{-1}x_{i}y_{i}+\frac{C^{-1}x_{i}x_{i}^{\prime }\beta^{r}}{1-h_{ii}^{r}}-\frac{C^{-1}x_{i}h_{ii}^{r}y_{i}}{1-h_{ii}^{r}}\\ =\beta^{r}-\frac{\left(1-h_{ii}^{r}\right)C^{-1}x_{i}y_{i}-C^{-1}x_{i}x_{i}^{\prime }\beta^{r}+h_{ii}^{r}C^{-1}x_{i}y_{i}}{1-h_{ii}^{r}}\\ =\beta^{r}-\frac{C^{-1}x_{i}\left(y_{i}-x_{i}^{\prime }\beta^{r}\right)}{1-h_{ii}}\\ =\beta^{r}-\frac{C^{-1}x_{i}{\hat{e}}_{i}^{r}}{1-h_{ii}}, \end{array}$$ (90)

where ${\hat{e}}_{i}^{r}=y_{i}-x_{i}^{\prime }\beta^{r}$ is the residual from the ridge regression-BLUP analysis obtained with the entire sample.

For leave-*d*-out the algebra is as with least-squares, producing as coefficient matrix

$${\left(X^{\prime }X+I\lambda -X_{\left[d\right]}^{\prime }X_{\left[d\right]}\right)}^{-1}=C^{-1}+C^{-1}X_{\left[d\right]}^{\prime }{\left[I-X_{\left[d\right]}C^{-1}X_{\left[d\right]}^{\prime }\right]}^{-1}X_{\left[d\right]}C^{-1},$$ (91)

and the right-hand sides are as before $X_{\left[-d,\right]}^{\prime }y_{\left[-d\right]}=X^{\prime }y-X_{\left[d\right]}^{\prime }y_{\left[d\right]}.$ Letting $H_{\left[d\right]}^{r}=X_{\left[d\right]}C^{-1}X_{\left[d\right]}^{\prime }$, algebra similar to the one producing (88) yields

$$\beta_{\left[-d\right]}^{r}=\beta^{r}-C^{-1}X_{\left[d\right]}^{\prime }{\left(I-H_{d}^{r}\right)}^{-1}{\hat{e}}_{\left[d\right]}^{r},$$ (92)

where ${\hat{e}}_{\left[d\right]}^{r}=y_{\left[d\right]}-X_{\left[d\right]}\beta^{r}$ is the d− dimensional residual from ridge regression BLUP applied to the entire sample.

### Appendix F: LOO and Leave-d-Out GBLUP

#### LOO GBLUP

In LOO CV, one seeks to estimate the mean of the predictive distribution of the left-out data point $E\left(y_{i}|y_{-i},H\right).$ Since $y_{i}=x_{i}^{\prime }\beta +e_{i}=g_{i}+e_{i}$ and $e_{i}∼N\left(0,\sigma_{e}^{2}\right)$ is independent of $y_{-i}$ one has

$$E\left(y_{i}|y_{-i},H\right)=E\left(g_{i}+e_{i}|y_{\left[-i\right]},H\right)=E\left(g_{i}|y_{\left[-i\right]},H\right).$$ (93)

More generally, we are interested in predicting all *n* genetic values $g=\left\{g_{i}\right\}.$ Since our zero-mean BLUP is interpretable as the mean of the conditional or posterior distribution [g|y,θ] (θ denotes $\sigma_{g}^{2}$ and $\sigma_{e}^{2}$ here), for p(.|.) denoting a density function, it follows that

$$p\left(g|y_{-i},\theta \right)\propto p\left(y_{-i}|g,\theta \right)p\left(g|\theta \right)\propto \frac{p\left(y|g,\theta \right)}{p\left(y_{i}|g,\theta \right)}p\left(g|\theta \right),$$ (94)

since $p\left(y|g,\theta \right)=p\left(y_{i}|g,\theta \right)p\left(y_{-i}|g,\theta \right),$ due to independence of residuals. The preceding posterior is Gaussian (given the dispersion parameters) because the prior and the sampling model are both Gaussian; hence, the mode and the median of this distributions are identical. Apart from an additive constant

$$\text{log}\left[p\left(g|y_{-i},\;\theta \right)\right]=\text{log}\;p\left(y|g,\theta \right)+\text{log}\;p\left(g|\theta \right)-\text{log}\;p\left(y_{i}|g,\theta \right),$$ (95)

or, explicitly,

$$\begin{array}{c} \text{log}\left[p\left(g|y_{-i},\theta \right)\right]=-\frac{1}{2\sigma_{e}^{2}}{\left(y-g\right)}^{\prime }\left(y-g\right)-\frac{1}{2\sigma_{g}^{2}}g^{\prime }G^{-1}g+\frac{1}{2\sigma_{e}^{2}}{\left(y_{i}-g_{i}\right)}^{2}\\ =-\frac{1}{2\sigma_{e}^{2}}\left[{\left(y-g\right)}^{\prime }\left(y-g\right)+\lambda g^{\prime }G^{-1}g-{\left(y_{i}-\delta_{i}^{\prime }g\right)}^{2}\right] \end{array}$$ (96)

where $\lambda =\frac{\sigma_{e}^{2}}{\sigma_{g}^{2}}$, and $\delta_{i}^{\text{*}}$ is a n×1 vector with a 1 in position *i* and 0’s elsewhere. The gradient of the log-posterior with respect to g is

$$\frac{\partial }{\partial g}\text{log}\left[p\left(g|y_{-i},\theta \right)\right]=-\frac{1}{2\sigma_{e}^{2}}\left[-2\left(y-g\right)+2\lambda G^{-1}g+2\delta_{i}^{\text{*}}\left(y_{i}-\delta_{i}^{\text{*}}g\right)\right];$$ (97)

note that $\delta_{i}^{\text{*}\prime }g=g_{i}$. Setting to zero yields the joint mode, *i.e.*, the BLUP of all *n* lines (but using $y_{-i}$) as the solution to the linear system

$$\left(C-\Delta_{i}\right){\tilde{g}}_{\left[-i\right]}=y-\delta_{i}^{\text{*}}y_{i},$$ (98)

where ${\tilde{g}}_{\left[-i\right]}$ (n×1) is the BLUP of g calculated with all observations other than i; $C=I+G^{-1}\lambda$ is the coefficient matrix used for calculating $BLUP\left(g\right)=\hat{g}=\left\{{\hat{g}}_{i}\right\}=$ $C^{-1}y$ using all data points; $\delta_{i}$ is as defined earlier , and $\Delta_{i}=\delta_{i}^{\text{*}}\delta_{i}^{\text{*}\prime }$ is an n×n matrix of $0^{\prime }s$ except for a 1 in position (i,i). Observe that

$${\tilde{g}}_{\left[-i\right]}=C^{-1}y-C^{-1}\delta_{i}^{\text{*}}y_{i}+C^{-1}\Delta_{i}{\tilde{g}}_{\left[-i\right]}=\hat{g}-C^{-1}\delta_{i}^{\text{*}}y_{i}+C^{-1}\Delta_{i}{\tilde{g}}_{\left[-i\right]}.$$ (99)

Further,

$$\begin{array}{l} C^{-1}\delta_{i}^{\text{*}}y_{i}=c^{i}y_{i},\\ C^{-1}\Delta_{i}{\tilde{g}}_{\left[-i\right]}=c^{i}{\tilde{g}}_{i}, \end{array}$$ (100)

where $c^{i}$ is the $i^{th}$ column of $C^{-1}=\left\{c^{ii}\right\}$, and ${\tilde{g}}_{i}$ is the $i^{th}$ element of ${\tilde{g}}_{\left[-i\right]}$. Thus

$${\tilde{g}}_{\left[-i\right]}-c^{i}{\tilde{g}}_{i}=\hat{g}-c^{i}y_{i}.$$ (101)

Inspection of the preceding expression reveals that element *i* of ${\tilde{g}}_{\left[-i\right]}$, that is, the genetic value of the observation left out in the LOO CV can be computed as

$${\tilde{g}}_{i}=\frac{1}{1-c^{ii}}\left({\hat{g}}_{i}-c^{ii}y_{i}\right);\;\;i=1,2,\ldots ,n.$$ (102)

If desired, the remaining n−1 elements of ${\tilde{g}}_{\left[-i\right]}$ can be calculated recursively as

$${\tilde{g}}_{j}={\hat{g}}_{j}-c^{ji}\left(y_{i}-{\tilde{g}}_{i}\right);\;\;j\neq i.$$ (103)

The observed predictive MSE is given by

$$PMSE\left(1\right)=\frac{1}{n}\sum_{i=1}^{n}{\left[y_{i}-\frac{1}{1-c^{ii}}\left({\hat{g}}_{i}-c^{ii}y_{i}\right)\right]}^{2}=\frac{1}{n}\sum_{i=1}^{n}{\left(\frac{y_{i}-{\hat{g}}_{i}}{1-c^{ii}}\right)}^{2}.$$ (104)

#### Leave-d-Out GBLUP

Consider next a leave-*d*-out setting. Assume that observations have been reordered such that phenotypes of members of the testing set are placed in the upper *d* positions in y, so $y^{\prime }=\left[y_{d},y_{\left[-d\right]}\right]$ and $g^{\prime }=\left[g_{d},g_{\left[-d\right]}\right].$ Thus

$$\begin{array}{c} \text{log}\left[p\left(g|y_{\left[-d\right]},\theta \right)\right]=-\frac{1}{2\sigma_{e}^{2}}{\left(y-g\right)}^{\prime }\left(y-g\right)-\frac{1}{2\sigma_{g}^{2}}g^{\prime }G^{-1}g+\frac{1}{2\sigma_{e}^{2}}{\left(y_{d}-g_{d}\right)}^{\prime }\left(y_{d}-g_{d}\right)\\ =-\frac{1}{2\sigma_{e}^{2}}\left[{\left(y-g\right)}^{\prime }\left(y-g\right)+\lambda g^{\prime }G^{-1}g-{\left(y_{d}-\Delta_{d}g\right)}^{\prime }\left(y_{d}-\Delta_{d}g\right)\right], \end{array}$$ (105)

where $\Delta_{d}=\left[\begin{array}{cc} I_{d} & 0_{d,n-d} \end{array}\right]$ is a d×n matrix partitioned into an identity d×d submatrix, and with a 0 as each element of the d×(n−d) partition. Hence

$$\frac{\partial }{\partial g}\text{log}\left[p\left(g|y_{\left[-d\right]},\theta \right)\right]=-\frac{1}{2\sigma_{e}^{2}}\left[-2\left(y-g\right)+2\lambda G^{-1}g+2\Delta_{d}^{\prime }\left(y_{d}-\Delta_{d}g\right)\right]$$ (106)

Setting the vector of differentials to 0, and rearranging

$${\tilde{g}}_{\left[-d\right]}={\left(C-\Delta_{d}^{\prime }\Delta_{d}\right)}^{-1}\left(y-\Delta_{d}^{\prime }y_{d}\right)={\left(C-\Delta_{d}^{\prime }\Delta_{d}\right)}^{-1}{\tilde{y}}_{d},$$ (107)

where ${\tilde{y}}_{d}$ is y with the *d* phenotypes in the testing set replaced by $0^{\prime }s$. Application of (79) produces

$${\left(C-\Delta_{d}^{\prime }\Delta_{d}\right)}^{-1}=C^{-1}-C^{-1}\Delta_{d}^{\prime }\left(I_{d}-\Delta_{d}C^{-1}\Delta_{d}^{\prime }\right)\Delta_{d}C^{-1}$$ (108)

Let now the inverse of the coefficient matrix for computing the BLUP of all individuals, or lines from all data points in y, be partitioned as

$$C^{-1}={\left(I+G^{-1}\lambda \right)}^{-1}=\left[\begin{array}{cc} C^{dd} & C^{d,-d}\\ C^{-d,d} & C^{-d,-d} \end{array}\right].$$ (109)

Then

$$\begin{array}{c} C^{-1}\Delta_{d}^{\prime }=\left[\begin{array}{cc} C^{dd} & C^{d,-d}\\ C^{-d,d} & C^{-d,-d} \end{array}\right]\left[\begin{array}{c} I\\ 0_{n-d,d} \end{array}\right]=\left[\begin{array}{c} C^{dd}\\ C^{-d,d} \end{array}\right],\\ \Delta_{d}C^{-1}\Delta_{d}^{\prime }=\left[\begin{array}{cc} I_{d} & 0_{d,n-d} \end{array}\right]\left[\begin{array}{cc} C^{dd} & C^{d,-d}\\ C^{-d,d} & C^{-d,-d} \end{array}\right]\left[\begin{array}{c} I\\ 0_{n-d,d} \end{array}\right] \end{array}$$ (110)

$$=\left[\begin{array}{cc} I_{d} & 0_{d,n-d} \end{array}\right]\left[\begin{array}{c} C^{dd}\\ C^{-d,d} \end{array}\right]=C^{dd},$$ (111)

$$\Delta_{d}C^{-1}=\left[\begin{array}{cc} C^{dd} & C^{d,-d} \end{array}\right]$$ (112)

Inspection of the form of (107) leads as BLUP predictor of phenotypes of individuals in the testing set to

$${\tilde{g}}_{d}={\left(I-C^{dd}\right)}^{-1}\left({\hat{g}}_{d}-C^{dd}y_{d}\right),$$ (113)

with CV prediction error

$${\tilde{\epsilon }}_{d}=y_{d}-{\tilde{g}}_{d}={\left(I-C^{dd}\right)}^{-1}\left(y_{d}-{\hat{g}}_{d}\right),$$

and realized predictive mean-squared error

$$\text{PMSE}\left(d\right)=\frac{{\overset{{∼}^{\prime }}{\epsilon }}_{d}{\tilde{\epsilon }}_{d}}{d}.$$ (114)

If desired, the BLUP predictors of the remaining n−d genotypes (based on $y_{\left[-d\right]}$) can be calculated as

$${\tilde{g}}_{\left[-d\right]}={\hat{g}}_{\left[-d\right]}-C^{-d,d}\left(y_{d}-{\tilde{g}}_{d}\right)$$ (115)

All preceding developments rest on assuming that exclusion of *d* observations does not modify *λ* in training sets in an appreciable manner.

### Appendix G: Cross-Validation Importance Sampling

The posterior expectation of the vector of marker effects is

$$E\left(\beta |y_{-i},H\right)=\frac{\int \beta p\left(\beta |y_{-i},H\right)d\beta }{\int p\left(\beta |y_{-i},H\right)d\beta }=\frac{\int \beta \frac{p\left(\beta |y_{-i},H\right)}{p\left(\beta |y,H\right)}p\left(\beta |y,H\right)d\beta }{\int \frac{p\left(\beta |y_{-i},H\right)}{p\left(\beta |y,H\right)}p\left(\beta |y,H\right)d\beta }$$ (116)

$$=\frac{\int w_{i}\left(\beta \right)\beta p\left(\beta |y,H\right)d\beta }{\int w_{i}\left(\beta \right)p\left(\beta |y,H\right)d\beta }=\frac{E_{\beta |y,H}\left[w_{i}\left(\beta \right)\beta \right]}{E_{\beta |y,H}\left[w_{i}\left(\beta \right)\right]},i=1,2,\ldots ,n.$$ (117)

Here, $w_{i}\left(\beta \right)=\frac{p\left(\beta |y_{-i},H\right)}{p\left(\beta |y,H\right)}$ is an “importance sampling” weight (Albert, 2009). Bayes theorem yields

$$w_{i}\left(\beta \right)=\frac{p\left(\beta |y_{-i},H\right)}{p\left(\beta |y,H\right)}=\frac{\frac{p\left(y_{-i}|\beta ,\sigma_{e}^{2}\right)p\left(\beta |H\right)}{p\left(y_{-i}|H\right)}}{\frac{p\left(y|\beta ,\sigma_{e}^{2}\right)p\left(\beta |H\right)}{p\left(y|H\right)}}=\frac{p\left(y|H\right)}{p\left(y_{-i}|H\right)}\frac{1}{p\left(y_{i}|\beta ,\sigma_{e}^{2}\right)},$$ (118)

and employing this form of $w_{i}\left(\beta \right)$ in (117) produces

$$E\left(\beta |y_{-i},H\right)=\frac{\int \frac{p\left(y|H\right)}{p\left(y_{-i}|H\right)}\frac{1}{p\left(y_{i}|\beta ,\sigma_{e}^{2}\right)}\beta p\left(\beta |y,H\right)d\beta }{\int \frac{p\left(y|H\right)}{p\left(y_{-i}|H\right)}\frac{1}{p\left(y_{i}|\beta ,\sigma_{e}^{2}\right)}p\left(\beta |y,H\right)d\beta }=\frac{\int \frac{1}{p\left(y_{i}|\beta ,\sigma_{e}^{2}\right)}\beta p\left(\beta |y,H\right)d\beta }{\int \frac{1}{p\left(y_{i}|\beta ,\sigma_{e}^{2}\right)}p\left(\beta |y,H\right)d\beta }.$$ (119)

The implication is that, given draws $\beta^{\left(s\right)},$ $\sigma_{e}^{2\left(s\right)}$ (s=1,2,…,S) from the full-posterior distribution, the posterior expectation (119) can be estimated as

$$\hat{E}\left(\beta |y_{-i},H\right)=\frac{\sum_{s=1}^{S}\frac{1}{p\left(y_{i}|\beta^{\left(s\right)},\sigma_{e}^{2\left(s\right)}\right)}\beta^{\left(s\right)}}{\sum_{s=1}^{S}\frac{1}{p\left(y_{i}|\beta^{\left(s\right)},\sigma_{e}^{2\left(s\right)}\right)}}=\sum_{s=1}^{S}w_{i,s}\beta^{\left(s\right)};\;\;i=1,2,\ldots ,n,$$ (120)

where

$$w_{i,s}=\frac{p^{-1}\left(y_{i}|\beta^{\left(s\right)},\sigma_{e}^{2\left(s\right)}\right)}{\sum_{s=1}^{S}p^{-1}\left(y_{i}|\beta^{\left(s\right)},\sigma_{e}^{2\left(s\right)}\right)};\;\;i=1,2,\ldots ,n;\;\;s=1,2,\ldots ,S.$$ (121)
